# Multifunctional Bamboo Based Materials Empowered by Multiscale Hierarchical Structures—A Critical Review

**DOI:** 10.1002/adma.202507844

**Published:** 2025-09-28

**Authors:** Yuxiang Huang, Juan Hu, Yahui Zhang, Yanglun Yu, Daihui Zhang, Qiumei Jing, Muhammad Wakil Shahzad, Saddick Donkor, Chi Chen, Chuan Wei Zhang, Ximin He, Ben Bin Xu, Shengbo Ge, Wenji Yu

**Affiliations:** ^1^ Research Institute of Wood Industry Chinese Academy of Forestry Haidian Beijing 100091 China; ^2^ National Key Laboratory for Development and Utilization of Forest Food Resources Institute of Chemical Industry of Forest Products Chinese Academy of Forestry Nanjing 210042 China; ^3^ Co‐Innovation Center of Efficient Processing and Utilization of Forest Resources College of Materials Science and Engineering Nanjing Forestry University Nanjing 210037 China; ^4^ School of Engineering, Physics and Mathematics Northumbria University Newcastle upon Tyne NE1 8ST UK; ^5^ Department of Materials Science and Engineering University of California, Los Angeles (UCLA) Los Angeles CA 90095 USA

**Keywords:** bamboo, hierarchical structure, multifunctional applications, species‐specific variation, sustainable materials

## Abstract

The global pursuit of carbon neutrality and sustainable alternatives to conventional materials has spurred intense interest in renewable biomass resources. Among these, bamboo stands out as a promising candidate due to its exceptional mechanical properties, rapid growth, and remarkable carbon sequestration capacity. This review critically examines the potential of bamboo‐derived materials for advanced multifunctional applications, focusing on tailoring their hierarchical structures and chemical versatility to achieve diverse performance characteristics. First, an in‐depth analysis is provided of the structure–property–function relationships of different bamboo components across multiple length scales, emphasizing the importance of species‐specific variations and innovative processing techniques. Second, recent advancements in the multifunctional properties of bamboo‐derived materials are highlighted for their potential applications in addressing critical societal challenges, including energy storage, flexible electronics, and biomedicine. Finally, the limitations of current approaches, including scalability and long‐term stability, are discussed and future research directions are proposed to unlock the full potential of sustainable bamboo materials for a circular bioeconomy.

## Graphical abstract

This review summarizes the latest progress in transforming renewable bamboo resources into high‐value functional materials. It emphasizes how to leverage bamboo's multiscale hierarchical structures to realize innovative applications in energy, construction, environment, and medicine, providing key insights for developing high‐performance bio‐based materials.

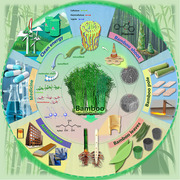

## 1 Introduction

The escalating global challenges of climate change, resource depletion, and environmental pollution request urgent and innovative solutions for sustainable development.^[^ ^1^ ^]^ Transitioning to a circular economy requires the exploration of renewable, environmentally friendly alternatives to conventional materials.^[^ ^2^, ^3^, ^4^ ^]^ Bamboo, as a bioresource material, offers unique advantages—including a rapid growth rate (up to 1 m per day), abundant resources (nearly 3.5 billion bamboo per year in China), high carbon sequestration capacity (17 tons of CO_2_ per hectare annually—four times that of tropical rainforests),^[^ ^5^, ^6^ ^]^ substantial biodiversity through erosion control and habitat provision to address critical societal needs across diverse sectors.

While bamboo‐based materials have played a role throughout human civilization, a comprehensive understanding of their structure–property relationships emerged relatively late Research on bamboo has progressed through three distinct chronological phases. i) Early (*pre‐2010s*), most work focused on exploring bamboo's potential as a sustainable material, identifying its high strength, rapid renewability, and lightweight properties.^[^ ^7^ ^]^ However, technical challenges arising from bamboo's intrinsic structural irregularity and anisotropy remained significant, which limited its widespread adoption. ii) *In the 2010s*, the research hotspot shifted toward achieving novel bamboo products by developing a variety of engineering methods to harness the structural integrity at millimeter to micrometer scale, such as laminated bamboo lumber (LBL),^[^ ^8^, ^9^ ^]^ which has excellent strength‐to‐weight ratio and sustainability. However, the high production costs and unresolved surface/interface issues continued to pose barriers to practical application. Subsequent studies^[^ ^10^, ^11^ ^]^ further advanced construction‐centric applications by providing baseline mechanical data for LBL. iii) *From the late 2010s to 2020*, the development of multifunctional applications and advanced processing techniques thrived the bamboo research along with a deeper understanding of bamboo structure at micro‐/meso‐scale. For instance, innovations in optical and thermal applications, such as the development of transparent bamboo,^[^ ^12^, ^13^ ^]^ demonstrated its potential to rival transparent wood and glass through delignification and resin impregnation techniques. At the same time, bamboo‐derived carbons were explored for energy storage and catalysis,^[^ ^14^, ^15^ ^]^ leveraging their high porosity, surface area, and heteroatom doping for applications in fuel cells, supercapacitors, and photothermal conversion. Thermal treatments^[^ ^16^, ^17^ ^]^ were utilized to facilitate hydrophilicity and interfacial bonding, thereby enhancing the mechanical strength and polymer compatibility of bamboo composites. iv) *In the 2020s*, several bionic and nanotechnology innovations have further expanded bamboo's potential by manipulating its chemical or physical structures at the nanoscale or below. Recent studies focus on bionic designs inspired by bamboo's natural structural gradients and nanotechnology applications, leading to breakthroughs in flexible electronics,^[^ ^18^ ^]^ advanced composites,^[^ ^19^, ^20^ ^]^ and optoelectronics.^[^ ^21^ ^]^

**Figure 1:**
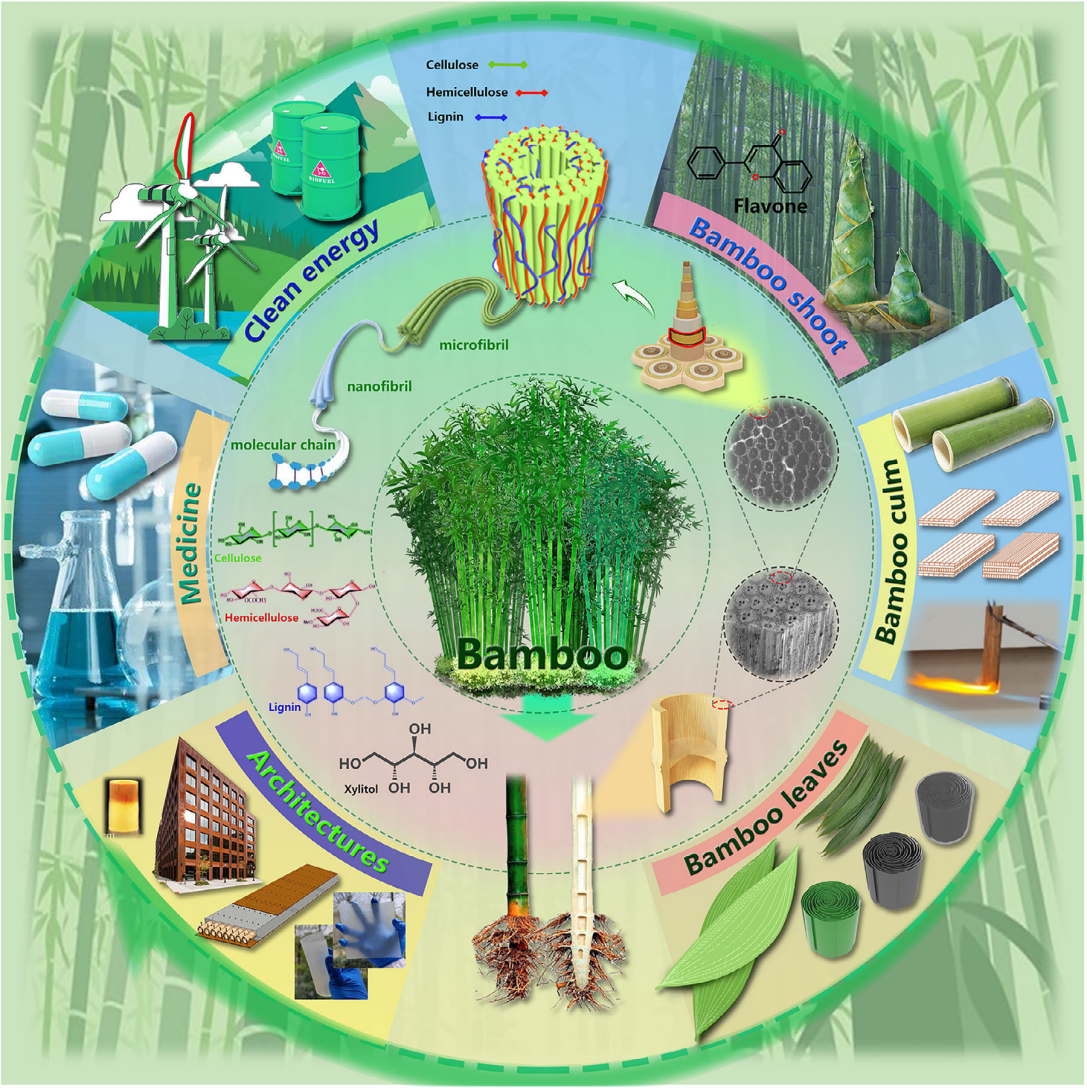
The illustration of the bamboo structure and its engineering applications.

This review aims to provide a comprehensive and critical analysis of recent progress in bamboo‐derived materials for advanced multifunctional applications (**Figure** **1**). Our work is distinguished by a strong emphasis on elucidating structure–property relationships across multiple length scales, from nanoscale interactions to macroscopic structures. The structure–property relationships are systematically mapped to guide the design and development of tailored applications. This review further highlights the significance of species‐specific variations and explores innovative processing strategies to optimize material performance. The application potential of bamboo is scoped in the field of energy storage, flexible electronics, and biomedicine, showcasing its ability to contribute to sustainable solutions for pressing global challenges. Moreover, a critical assessment of current limitations is conducted, and future research directions are outlined to lead the future development of high‐performance bamboo materials. Specifically, the subsequent sections of this review will detail the physical structure and chemical composition of bamboo (Section 2), explore its diverse range of functional materials and devices (Section 3), discuss the application of machine learning and artificial intelligence in this field (Section 4), examine the implications for a circular economy (Section 5), and conclude with a perspective on opportunities and challenges (Section 6).

## 2 Multiscale Structure and Structural Characteristics of Bamboo

**Figure 2:**
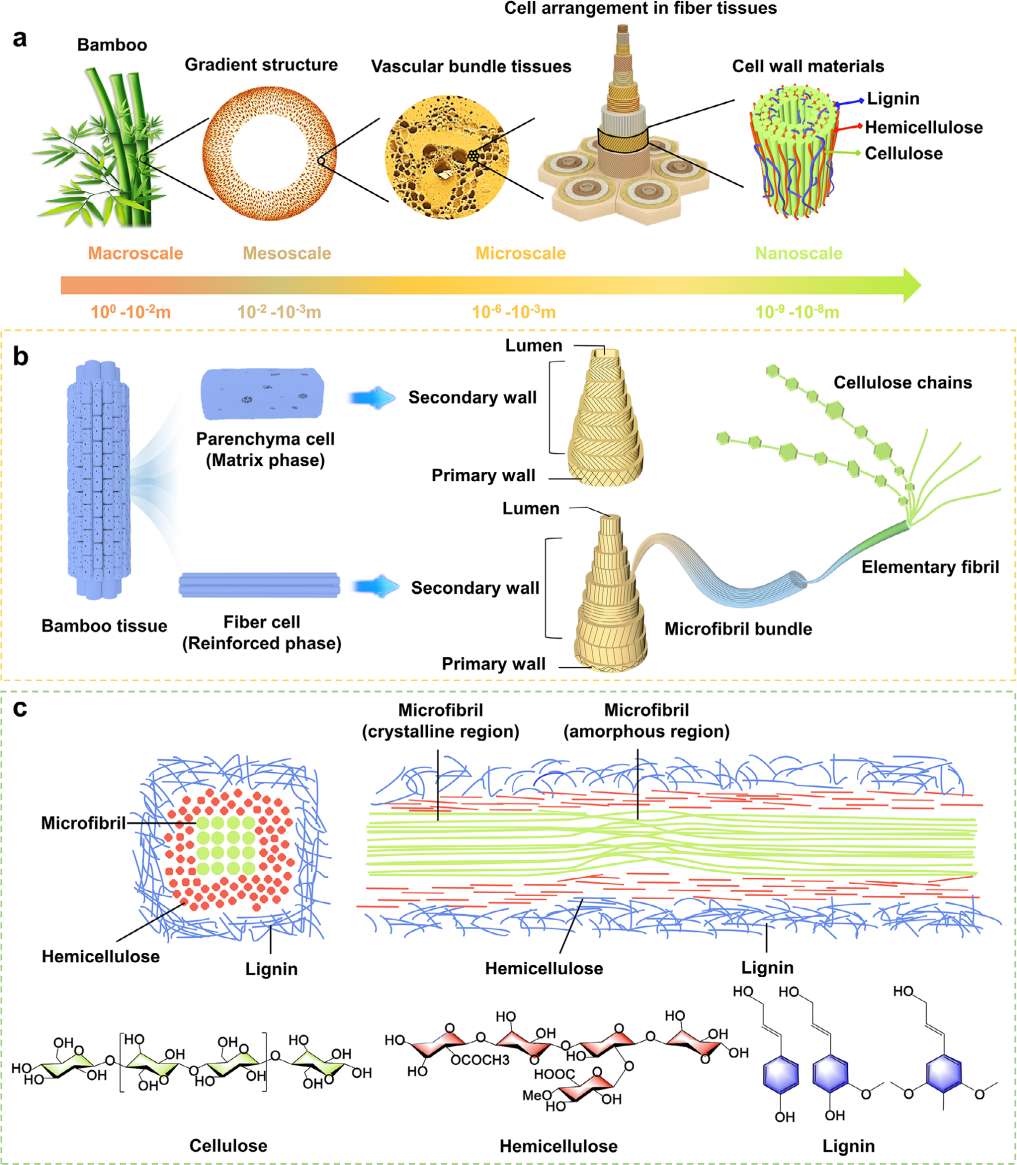
a) Hierarchical structure of bamboo culms at multiple length scales. b) Anatomical structure of bamboo at the nanoscale and microscale. c) Chemical composition of bamboo in cross section and longitudinal direction.

Bamboo is a gradient biomaterial with structural features that span from the macroscale to the nanoscale (**Figure** **2**a). At the macroscale (centimeter scale), it demonstrates a remarkable hollow multi‐node structure. At the mesoscale (millimeter scale), it exhibits a distinctive gradient structure, where the vascular bundles’ volume fraction diminishes from the outer to the inner regions, reflecting their functional distribution, wherein the volume fraction of vascular bundles progressively decreases. At the microscale (micrometer scale), bamboo consists of a two‐phase composite architecture, where vascular bundle fibers serve as reinforcement and thin‐walled cells form the matrix, arranged in both series and parallel configurations. At the nanometer scale, bamboo is composed of a two‐phase structure, in which cellulose acts as the reinforcing phase, embedded within a matrix of lignin and hemicellulose.

### 2.1 Cell and Cell Wall Architecture

Bamboo exhibits a distinctive island‐chain structure, characterized by rigid vascular bundles (VBs) and flexible parenchyma tissues.^[^ ^22^ ^]^ These VBs are composed of bamboo fibers (BFs) bound by intercellular layers and include a fiber sheath and conduits for transporting water and nutrient,^[^ ^23^ ^]^ as well as phloem tissue comprising sieve tubes and companion cells. BFs, which account for 40% of the culm's structure^[^ ^24^ ^]^ and feature elongated, tapering ends aligned longitudinally, provide essential mechanical support.^[^ ^25^ ^]^ The vessels in bamboo play a vital role in the processes of growth and development by facilitating the transportation of water and inorganic salts.^[^ ^26^ ^]^ Also, sieve tubes and companion cells are instrumental in the longitudinal permeation within bamboo. VBs can be classified into different types based on the arrangement and morphology of fiber sheaths.^[^ ^27^ ^]^ In general, the VBs near the greener side of bamboo culm are smaller and denser, whereas those near the yellow side are larger and more scattered. Parenchyma tissues, accounting for 52% of the bamboo material, consist of two types: basic parenchyma tissues and vascular bundle‐associated parenchyma tissues. The former includes elongated and short cells, while the latter are interspersed among the former, enhancing elasticity and facilitating load transfer within the culm. Additionally, these vascular bundle‐associated thin‐walled tissues store and transport nutrients because of the starch grains and numerous pit pores on their cell walls.^[^ ^28^ ^]^

The cell walls of bamboo wood are intricately structured, composed of primary and alternating wide and narrow secondary walls (Figure 2b).^[^ ^29^ ^]^ This multi‐layered, hierarchical arrangement undergoes continuous evolution as the bamboo grows and experiences lignification.^[^ ^30^ ^]^ The unique structure of cell walls plays a critical role in influencing bamboo's growth, water transportation capabilities, protection mechanisms, and mechanical properties.^[^ ^31^ ^]^ It is particularly influential in imparting high tensile strength and toughness to bamboo materials, where the mechanical strength is significantly affected by the arrangement of microfibrils. In BFs, the primary wall microfibrils are disorganized, adapting to the needs of cell elongation and growth while the secondary wall microfibrils are more oriented, enhancing the mechanical strength of cell walls.^[^ ^32^ ^]^ But in thin‐walled cells, the cell wall features a multilayer structure, albeit with a loose structure.^[^ ^33^ ^]^ Overall, a smaller average angle of microfibrils in the cell wall is associated with better mechanical performance.^[^ ^31^ ^]^

Bamboo's structures are shaped by key anatomical features, including vascular bundle density, cell wall thickness, fiber morphology, and fiber sheath distribution. These traits vary significantly across species, impacting the material's mechanical strength. For instance, Hartono et al.^[^ ^34^ ^]^ analyzed six bamboo species from Sumatera Island and observed notable differences in VBs density and fiber wall dimensions, both of which are closely linked to mechanical performance. Similarly, Abdullah et al.^[^ ^35^ ^]^ examined thirteen Malaysian species and found that species such as *Gigantochloa thoii* and *Gigantochloa scortechinii* exhibited higher VBs density, which contributed to superior modulus of rupture (MOR) and tensile strength. These findings are supported by Portal‐Cahuana et al.,^[^ ^36^ ^]^ who documented anatomical differences among three Peruvian species, highlighting how variations in fiber proportions and VBs characteristics significantly influence compressive strength and bending performance.

### 2.2 Chemical Composition

The primary constituents of bamboo culm tissue are cellulose (40–55%), hemicellulose (15–30%), and lignin (20–30%), predominantly found in the cell walls (Figure 2c). These elements define the structural and functional properties of bamboo culm cell walls, influencing their processing and utilization. Minor components such as soluble polysaccharides, proteins, resins, tannins, and ash, are mainly located within the lumen of bamboo culm cells. The chemical composition varies with age, culm position, and treatment processes. A common finding is that lignin content increases with age while hemicellulose decreases, making mature bamboo (3–5 years) structurally superior but less suitable for enzymatic processing.^[^ ^37^, ^38^ ^]^

#### 2.2.1 Cellulose

Cellulose is a high‐molecular‐weight linear homopolymer composed of glucose units linked by β‐1,4‐glycosidic bonds,^[^ ^39^ ^]^ forming the structural backbone of the cell wall and contributing to bamboo's strength and durability. Bamboo cellulose is rich in hydroxyl groups that enable extensive hydrogen bonding both within and between polymer chains, and its high degree of polymerization, typically ranging from 8000 to 12 000, further contributes to bamboo's mechanical properties.

#### 2.2.2 Hemicellulose

Hemicellulose comprises short, linear polymers of xylose with side chains, where structure varies based on bamboo species and growth conditions. It plays a crucial role in binding to other cell wall components, and enhancing structural integrity. These compounds are connected by covalent ester ether bridges, diester crosslinks and hydrogen bonds. Its degree of polymerization is much lower than cellulose, typically around 100–200. The structure of hemicellulose, along with its hydrophilic hydroxyl and carbonyl groups, contributes to bamboo's moisture absorption and dimensional stability.

#### 2.2.3 Lignin

Lignin is a complex aromatic polymer composed of phenyl‐propane units (H, G, and S units), interconnected in a specific ratio, through biosynthetic pathways. The molecular weight of bamboo lignin can vary widely depending on the species and extraction method, but typically falls within the range of 2000 to 15 000 g mol^−1^.^[^ ^40^ ^]^ It surrounds the carbohydrate components consisting of cellulose and hemicellulose, providing cell wall impermeability and enhancing the mechanical strength of bamboo materials.

High cellulose content enhances mechanical properties such as tensile strength and durability, making species like *Phyllostachys pubescens* and *Bambusa arundinacea* ideal for structural and tensile‐critical uses.^[^ ^34^ ^]^ Conversely, lignin‐rich species (*Dendrocalamus strictus, Dendrocalamus asper*) exhibit superior compressive strength and thermal stability due to lignin's role in stiffening cell walls.^[^ ^41^, ^42^ ^]^ Studies like Yeh and Yang^[^ ^41^ ^]^ and Rizal et al.^[^ ^43^ ^]^ confirm the strong correlation between lignin content and thermal decomposition resistance, identifying lignin‐rich bamboo as more suitable for bioenergy applications. Hemicellulose, however, presents a trade‐off. Species with high hemicellulose content tend to exhibit greater moisture absorption and swelling, as reported in studies by Sulaiman et al.^[^ ^44^ ^]^ and Zakikhani et al.^[^ ^45^ ^]^ This highlights hemicellulose as a limiting factor for long‐term dimensional stability in humidity‐prone environments, particularly for lower‐density species like *Bambusa vulgaris*.

### 2.3 Structural Characteristics of Bamboo

**Figure 3:**
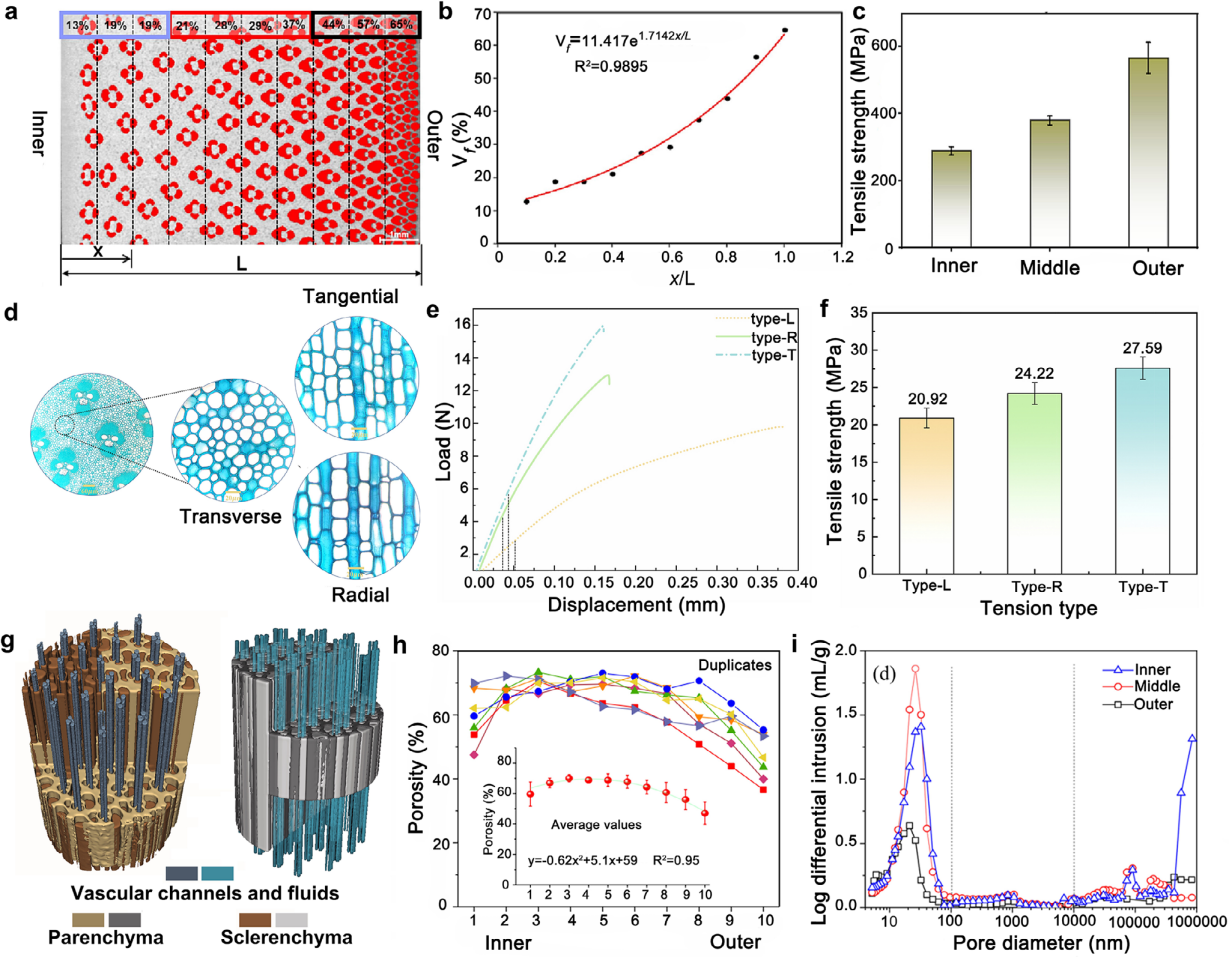
a) Fiber volume fraction distribution in the radial direction. Reproduced with permission.^[^ ^47^ ^]^ Copyright 2023, Elsevier B.V. b) distribution relation between the volume fraction of fibers (*V*_f_) and the location parameter (*x*/L) of bamboo in the radial direction. Reproduced with permission.^[^ ^47^ ^]^ Copyright 2023, Elsevier B.V. c) Bamboo fiber bundle tensile strength on the radial direction of the bamboo stem. Reproduced with permission.^[^ ^47^ ^]^ Copyright 2023, Elsevier B.V. d) Morphology of bamboo parenchyma cells. Reproduced with permission.^[^ ^54^ ^]^ Copyright 2023, Springer Nature. e,f) Longitudinal, radial, and tangential tensile properties of parenchyma tissue. Reproduced with permission.^[^ ^54^ ^]^ Copyright 2023, Springer Nature. g) µCT 3D images of natural bamboo show the vascular channels for fluid transportation imbibed into sclerenchyma and parenchyma tissues. Reproduced with permission.^[^ ^56^ ^]^ Copyright 2023, Elsevier B.V. h) Overall porosity of ten radial bamboo pieces. Reproduced with permission.^[^ ^55^ ^]^ Copyright 2023, Springer Berlin Heidelberg. i) The differential pore size distribution of bamboo. Reproduced with permission.^[^ ^55^ ^]^ Copyright 2023, Springer Berlin Heidelberg.

The remarkable performance of bamboo originates from its sophisticated hierarchical architecture, which spans from the macroscopic culm down to the nanoscale cell wall. This intricate design dictates its mechanical anisotropy, fluid transport efficiency, and surface characteristics. A fundamental understanding of these structure‐property relationships is paramount for the rational design and optimization of advanced bamboo‐based materials (**Figure** **3**).

#### 2.3.1 Functionally Graded Fiber‐Matrix Architecture

The fabric structure of bamboo primarily refers to its natural hierarchical organization as fiber‐reinforced composites with longitudinally aligned VBs embedded in a parenchyma matrix. The fiber sheaths surrounding the VBs are the primary source of bamboo's high tensile strength and stiffness. This is directly evidenced by a pronounced radial density gradient, where the volume fraction of VBs increases exponentially from ≈13% on the inner side to over 60% on the outer side (Figure 3a,b).^[^ ^46^ ^]^ Correlating with this, the tensile strength of the fiber bundles themselves increases significantly from under 300 MPa in the inner region to over 500 MPa in the outer region (Figure 3c).^[^ ^47^ ^]^ This functionally graded structure strategically places the strongest components where bending stresses are highest, making bamboo a highly efficient natural structural material.^[^ ^48^ ^]^ In contrast, the softer parenchyma tissue acts as a matrix, providing a crucial buffering role. Its lower strength means that cracks tend to propagate within the parenchyma, which reduces processing resistance and contributes to bamboo's excellent toughness.^[^ ^49^ ^]^

#### 2.3.2 Anisotropic Structure

Bamboo's anisotropic structure, stemming from the non‐uniform distribution and orientation of its hierarchical constituents, profoundly influences its mechanical properties. Consequently, its mechanical properties vary significantly with direction. The longitudinal direction exhibits the highest tensile strength and stiffness,^[^ ^50^ ^]^ often an order of magnitude greater than in the radial or tangential directions.^[^ ^51^ ^]^ For example, the longitudinal Young's moduli of Moso bamboo is between 8.49 and 32.49 GPa, whereas its transverse modulus is typically around 2–5 GPa.^[^ ^52^, ^53^ ^]^ Additionally, research has also focused on the parenchyma cell level, revealing the microstructural basis of bamboo's anisotropy mechanism (Figure 3d). As shown by tensile tests on parenchyma tissue (Figure 3e,f), its strength is highest in the tangential direction (27.59 MPa), followed by the radial (24.22 MPa), and is weakest in the longitudinal direction (20.92 MPa). This distinct property of the matrix tissue is believed to be a key mechanism that dictates the zigzag crack propagation path observed during bamboo failure, thereby enhancing its overall fracture toughness.^[^ ^54^ ^]^ This inherent anisotropy is a critical design consideration, making bamboo ideal for unidirectional load‐bearing applications, while also necessitating strategies like lamination or densification to overcome its transverse weaknesses.

#### 2.3.3 Hierarchical Porosity and Anisotropic Fluid Transport

Bamboo possesses an intrinsic, multi‐scale porous network (Figure 3g) that is fundamental to its lightweight nature, dimensional stability, and processability. This porosity is substantial, typically ranging from 60% to 80% across the culm wall, with a slight decreasing trend observed from the inner to the outer region (Figure 3h).^[^ ^55^ ^]^ Pore size distribution analysis reveals at least two distinct populations (Figure 3i): macropores with diameters exceeding 10 000 nm, which correspond to the large vessel lumens, and a significant fraction of mesopores in the 20–100 nm range, attributed to the innate porosity within cell walls and parenchyma tissue.

This porous architecture forms a network of continuous, interconnected tunnels that govern anisotropic fluid transport. As visualized by µCT imaging (Figure 3g), the large vascular channels serve as the primary conduits for rapid, long‐range fluid flow via capillary action.^[^ ^56^ ^]^ These channels are embedded within two distinct tissue types: the dense fibers and the more porous parenchyma matrix. The material's overall hygroscopic behavior is therefore highly dependent on this internal structure. The parenchyma, with its higher porosity and less lignified walls,^[^ ^57^, ^58^ ^]^ acts as the primary site for moisture absorption and storage, while the less permeable sclerenchyma provides structural support.

The dual nature of this porous network presents both opportunities and challenges. On one hand, it is highly beneficial for creating lightweight materials and facilitating the impregnation of resins or functional agents during composite manufacturing. On the other, these voids can act as pathways for crack propagation, potentially compromising mechanical integrity. Consequently, understanding and engineering this hierarchical porosity—from controlling the internal surface chemistry via treatments to optimizing transport pathways—is a key strategy for developing advanced bamboo‐based materials with tailored performance.

## 3 Functional Materials and Devices Derived from Bamboo

By harnessing the structural characteristics of bamboo, sustainable multifunctional materials and devices can be developed and bamboo resource utilization for high‐value applications can be maximized. Currently, bamboo is utilized at various scales, ranging from the macroscale to the nanoscale and encompassing original bamboo, bamboo walls, bamboo cells, bamboo cell walls, and the three primary components of bamboo.

### 3.1 Conventional Bamboo‐Based Materials

**Figure 4:**
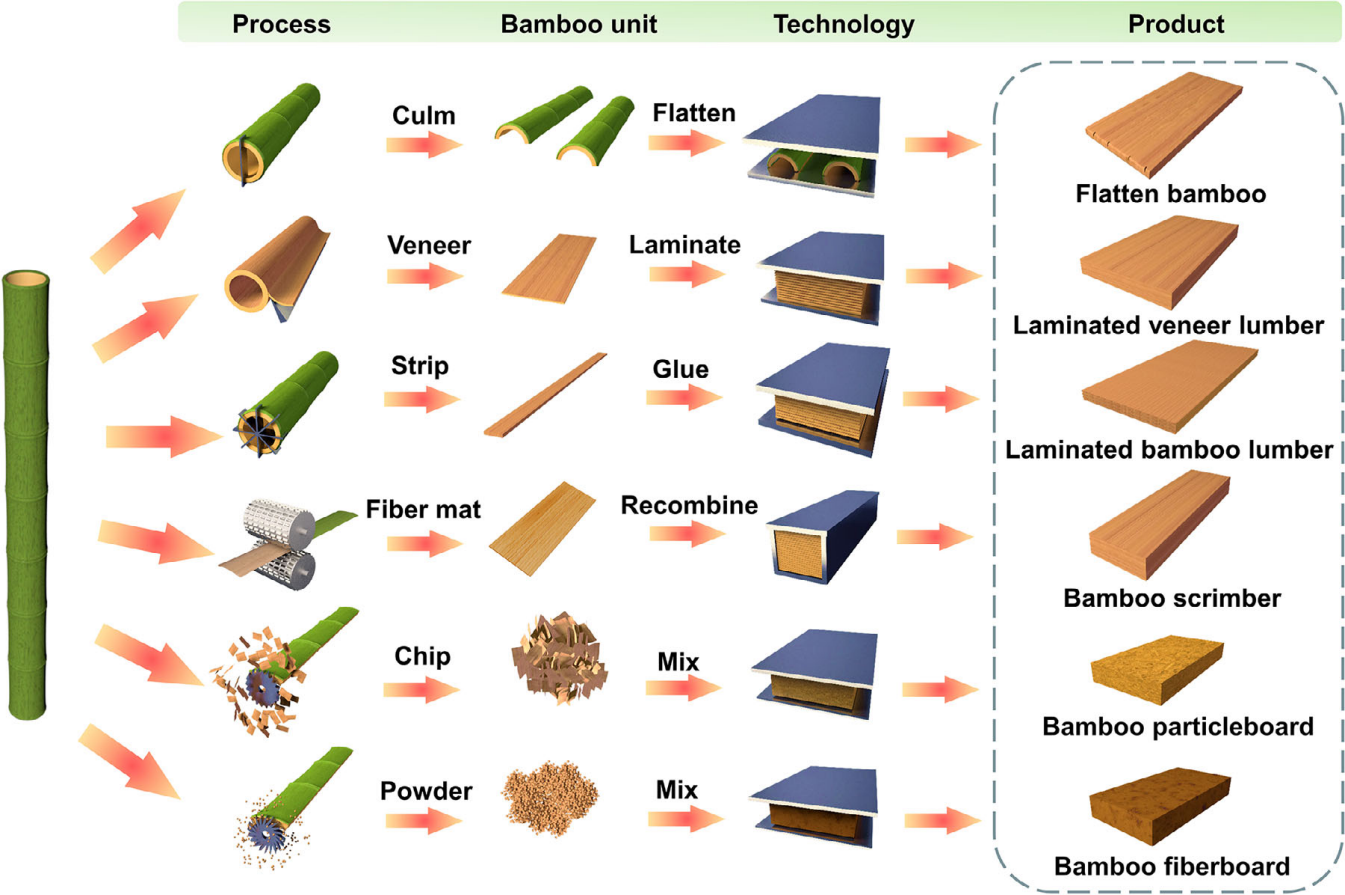
Schematic diagram of the preparation process of bamboo‐based panels composed of different bamboo units.

Conventional bamboo‐based materials include a series of bamboo‐based panels suitable for application in construction and engineering. These materials include flattened bamboo, laminated bamboo, scrimber, particleboards, and fiberboards (**Figure** **4**). They are made through processes that flatten or rotary‐cut bamboo culms into veneers, slice and plane bamboo into strips or pieces, and crush it into shavings or powders. Subsequently, the adhesive is applied, followed by hot‐pressing to produce bamboo‐based panels. These materials mitigate the inherent anisotropy, nonuniformity, and cracking susceptibility of bamboo, offering a performance that rivals or surpasses that of raw bamboo.^[^ ^8^ ^]^ The mechanical properties of these bamboo‐based panels are highly dependent on a combination of processing parameters and material characteristics, including densification level, resin content, and the architecture of the reconstituted fiber network, as summarized in **Table** **1**. Significantly, they have been successfully produced on a large industrial scale. For now, laminated bamboo lumber (LBL) and bamboo scrimber are the predominant engineering materials derived from bamboo.^[^ ^59^ ^]^

**Table 1:** Typical mechanical properties of different bamboo‐based panels.

Panel type	Process highlights	Density [g cm^−^^3^]	Modulus of rupture [MOR, MPa]	Modulus of elasticity [MOE, GPa]	Key reason for performance difference	Refs.
Flatten bamboo	Culms flatten under saturated steam heat treatment	0.6–0.7	65–75	13–17	Minimal processing, preserves natural structure	[60]
Laminated veneer lumber	Rotary‐cut veneers laminate with adhesive	0.6–0.75	60–160	5–12	Veneer quality and adhesive bond are critical	[61]
Laminated bamboo lumber (LBL)	Strips laminated with adhesive	0.6–0.8	80–150	8–12	Preserves fiber alignment; properties depend on lamination	[62]
Bamboo scrimber	Fiber mat recombined under high pressure	1.0–1.35	150–350	15–28	Extremely high density and fiber content; reconstituted structure	[63, 64]
Bamboo particleboard	Particles bonded with resin	0.6–0.75	13–30	1.3–4	Random particle orientation; low structural integrity	[65]
Bamboo fiberboard	Fibers hot‐pressed (often without synthetic resin)	0.7–0.8	11–45	2.1–5	Relies on lignin/fiber bonding; lower density than scrimber	[66]

LBL, a versatile composite material manufactured by bonding flat bamboo strips into engineered panels, has gained attention for its well‐defined mechanical properties and ease of use, making it comparable to structural timber and laminated veneer lumber for construction applications.^[^ ^9^ ^]^ It has three typical structures: horizontal, vertical, and multilayer cross‐laminated. Among these, vertical structures exhibit superior mechanical properties, making them suitable for use in diverse structural components. Considering the yield of the bamboo tubes, the length of the bamboo strips generally does not exceed 3 m. Therefore, when a large‐span LBL is required, it needs to be lengthened. Traditional finger lengthening significantly reduces the strength of LBL from 124 to 53 MPa.^[^ ^67^ ^]^ Hence, recent research has focused on lengthening bamboo strips and dispersing joint positions to minimize mechanical property loss. The joint quality of bamboo strips has a significant impact on the mechanical properties of bamboo laminated timber. Three bamboo strip lengthening methods are commonly used: butt joint, finger joint, and buckle‐type joint.^[^ ^68^ ^]^ Although the butt joint is relatively easy to process, its assembly is complex. In contrast, the lengthening method using buckle‐type joints requires specialized cutting tools but enables bamboo strip extension without adhesives. Considering the bamboo processing challenges and gluing difficulties, the buckle‐type joint is an ideal joint form. Currently, LBL produced with buckle‐type joints can extend over 10 m, offering unmatched size flexibility compared to other engineered materials. This makes it a versatile choice for applications such as car floor panels and furniture.

Bamboo scirmber represents a next‐generation engineered bamboo composite, distinguished by an innovative manufacturing route that deviates significantly from traditional methods. The process involves cutting, splitting, mechanical loosening, drying, and high‐pressure compaction.^[^ ^69^ ^]^ The key step is the mechanical loosening (or defibering) process, which sets it apart from conventional veneer or strip‐based lamination techniques. Instead of pre‐removing the outer (green) and inner (yellow) layers—which are rich in waxes and silica that have historically acted as barriers to adhesive bonding—the entire bamboo culm is processed. Specialized equipment transforms the culm directly into a three‐dimensional mat of longitudinally interconnected fiber bundles. This approach dramatically increases the material utilization rate to over 90%. These engineered fiber mats, with bundle widths controlled within the 30–300 µm range, not only retain the bamboo's native axial fiber alignment but also feature an expanded pore network and a higher specific surface area.^[^ ^70^ ^]^ This enhanced porosity facilitates deep and uniform adhesive penetration during impregnation and ensures the formation of robust, solid adhesive bonds upon curing. Consequently, the final bamboo scrimber product exhibits a remarkable density, often exceeding 1.2 g cm^−^^3^ superior adhesive bonding directly translates into outstanding macroscopic properties. The resulting bamboo scrimber exhibits not only high strength and stiffness but also exceptional dimensional stability. For instance, its thickness expansion rate after 24 h of water immersion can be as low as 0.4%, far surpassing the requirements of standards like EN13329 (which allows up to 20%). Furthermore, by adjusting parameters such as resin type and content, its water resistance and mechanical properties can be precisely tailored.^[^ ^71^ ^]^ These elite performance characteristics have enabled bamboo scrimber's extensive application in demanding fields, including durable outdoor flooring, high‐end furniture, architectural decoration, and even structural components for wind turbine blades.

These conventional materials not only take advantage of bamboo's high strength‐to‐weight ratio but also expand its usability into engineered forms that standardize its properties and dimensions. However, these materials are still constrained by challenges such as high costs, difficulty in connection techniques, and the intensive use of adhesives.

### 3.2 Tuning Bamboo Walls to Synthesize Materials

The hierarchical structure of bamboo, featuring VBs embedded in a parenchyma matrix with lignocellulosic walls, allows extensive tuning through chemical, thermal, and mechanical processes to yield high‐performance and functional materials.

#### 3.2.1 Bamboo‐Based Functional Composite Materials

Advanced bamboo‐based composites now play a critical role in creating high‐performance, multifunctional products. Functional composites derived from bamboo include high‐strength materials, flame‐retardant composites, antibacterial materials, and electromagnetic shielding composites, offering solutions for diverse applications like construction, biomedical devices, and electronics.

**Figure 5:**
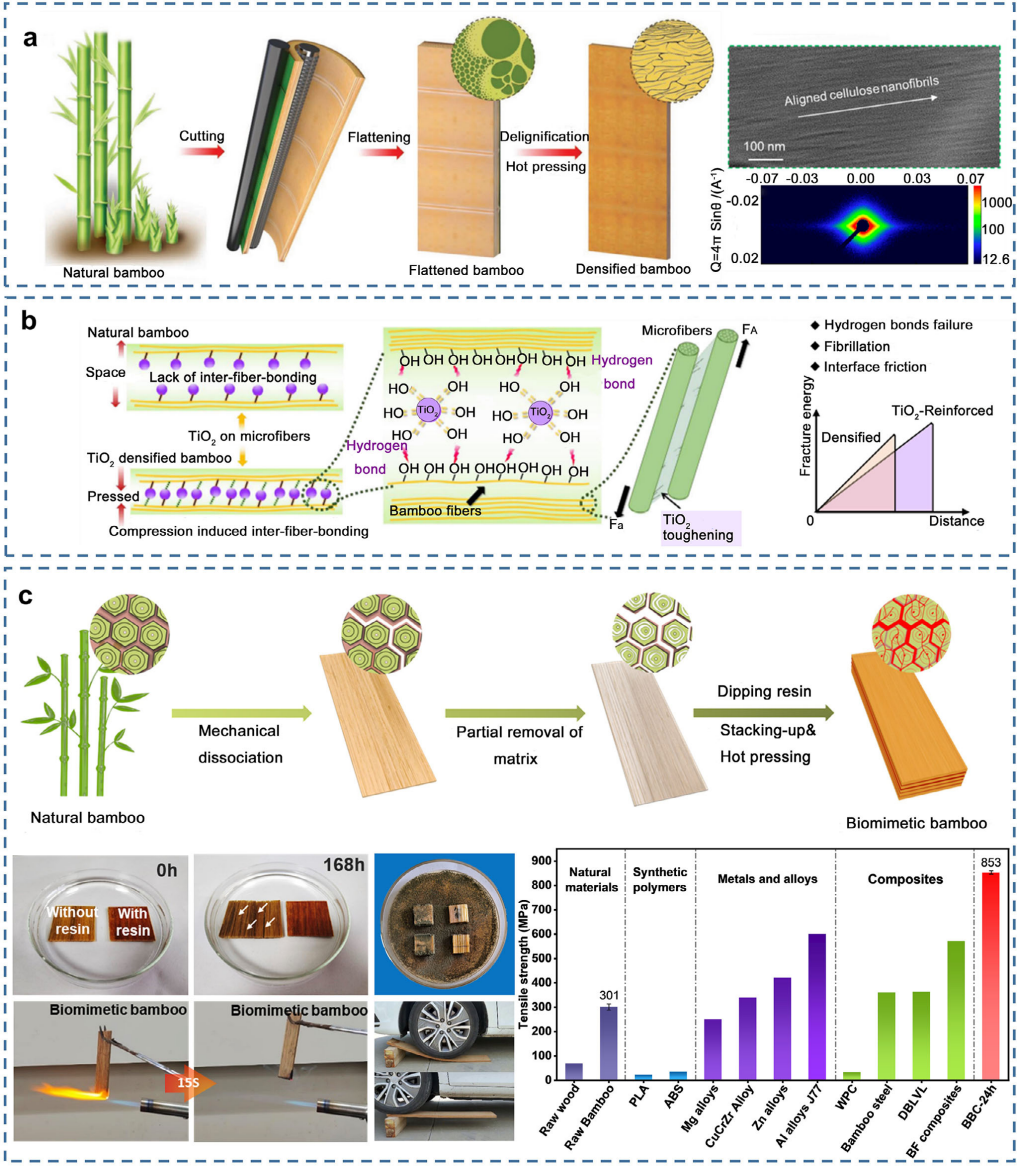
a) A schematic diagram of ultra‐strong bamboo materials, and its microfibrils arrangement orientation. Reproduced with permission.^[^ ^23^, ^73^ ^]^ Copyright 2020, Wiley‐VCH, American Chemical Society. b) The bonding mode and strengthening mechanism of TiO_2_ with natural bamboo and densified bamboo. Reproduced with permission.^[^ ^74^ ^]^ Copyright 2023, Springer Nature. c) The preparation process of biomimetic bamboo with good dimensional stability, flame retardant, mildew resistance and load‐bearing properties and comparison with other materials (natural materials, synthetic polymers, metal alloys, etc.) in tensile strength. Reproduced with permission.^[^ ^75^ ^]^ Copyright 2023, Elsevier B.V.

***High‐strength bamboo‐based composite*** is one of the most extensively studied material, focused on the applications in construction, automotive, and aerospace industries.^[^ ^72^ ^]^ They are normally fabricated via top‐down strategy involving processes like delignification, densification, resin impregnation, and hot‐pressing (**Table** **2**). Studies have demonstrated that partial removal of lignin and hemicellulose, followed by mechanical compression under heat, aligns cellulose fibers and increases bonding strength through hydrogen bonding.^[^ ^23^, ^73^ ^]^ The resulting densified bamboo composites possess impressive tensile strength (up to 1 GPa) and toughness (9.74 MJ m^−^^3^), rivaling steel and many commercial materials (**Figure** **5**a). Further research involved modifying bamboo by TiO_2_ modification and densification under pressure, thereby enhancing its oxidation and hydrogen bonding.^[^ ^74^ ^]^ This can potentially increase the toughness of composite bamboo materials via crack deflection and energy dissipation (Figure 5b). Inspired by the human spinal neural network, Hu et al. utilized phenolic resin to form a neural network‐like structure between and within bamboo layers, firmly controlling the compressed bamboo cells and thus allowing the development of high‐strength and large‐sized bamboo composites (Figure 5c).^[^ ^75^ ^]^ While internal hydrogen bonding provides environmentally friendly advantages without chemical additives, current research demonstrates significant limitations in stability under humidity variations. In comparison, chemical crosslinking enhances stability but potentially introduces toxicity issues, reducing material sustainability. Existing studies lack systematic comparative analysis, particularly quantitative comparisons of long‐term dimensional stability, mechanical property retention rates, and environmental compatibility. Future development requires innovative fixation methods that simultaneously satisfy high strength, environmental stability, and sustainability requirements, rather than addressing these challenges in isolation.

**Table 2:** Preparation methods and properties of high‐strength bamboo or bamboo composites.

Treatment methods for bamboo material	Bonding methods	Density [g cm^−3^]	Tensile strength [GPa]	Bending strength [GPa]	Refs.
Chemical removal of lignin and hemicellulose	Hydrogen bonding	1.35	1.05 GPa	0.40 GPa	[73]
Steam delignification and cellulose acetate deacetylation	Hydrogen bonding	1.35	1.1 GPa	–	[76]
Delignification and alginate impregnation	Dual cross‐linking	1.3	1.12 GPa	0.68 GPa	[77]
Partial delignification and microwave heating	Hydrogen bonding	1	0.56 GPa	0.25 GPa	[23]
Chemical removal of lignin and hemicellulose and epoxy resin impregnation	Hydrogen bonding and chemical bonding	1.35	0.41 GPa	0.51 GPa	[78]
Delignification and in situ hydrothermal synthesis of and TiO_2_	Hydrogen bonding	1.34	0.39 GPa	0.42 GPa	[74]
Mechanical dissociation, partial delignification and phenolic resin impregnation	Hydrogen bonding and physical adhesive nails	1.35	0.85 GPa	0.52 GPa	[75]

Beyond high‐strength applications, bamboo composites are being engineered with a diverse array of advanced functionalities, including flame retardancy, antibacterial efficacy, and electromagnetic interference (EMI) shielding. These next‐generation materials are typically realized by integrating functional additives—ranging from chemical agents to nanomaterials—that leverage bamboo's intrinsic hierarchical architecture.

For flame retardancy, diverse strategies are employed, from bulk modification to surface engineering, to mitigate the inherent flammability of bamboo. Bulk modification, such as treating the delignified cellulose scaffold with boric acid prior to epoxy infiltration, creates internal cross‐links that promote charring.^[^ ^79^ ^]^ This results in a composite with a limiting oxygen index of 26.5% and a 63% reduction in peak heat release rate, while also enhancing mechanical strength. In situ mineralization offers another route, where precipitating CaCO_3_ nanoparticles directly within the porous parenchyma cells creates a stable, non‐combustible inorganic network.^[^ ^80^ ^]^ Alternatively, applying surface coatings, such as those containing hexagonal boron nitride, provides a protective barrier that can delay ignition time by 56% and significantly suppress total smoke production, offering effective protection without altering the bulk material.^[^ ^81^ ^]^

In the realm of antibacterial materials, the porous bamboo scaffold is functionalized primarily through the in situ synthesis of metal‐organic frameworks (MOFs) or the deposition of inorganic nanoparticles. One sophisticated approach involves growing copper‐based MOFs like MOF199 directly onto fiber surfaces.^[^ ^82^ ^]^ Chemical pre‐treatments create nucleation sites, yielding a dense coating that reduces *S. aureus* colonies from 8.98 to 2.08 CFU cm^−2^. A more common strategy utilizes inorganic nanoparticles; for instance, ZnO/graphene oxide nanocomposites hydrothermally grown within bamboo exhibit broad‐spectrum efficacy against both Gram‐positive (*B. subtilis*) and Gram‐negative (*E. coli*) bacteria.^[^ ^83^ ^]^ Similarly, a dense layer of in situ grown silver nanoparticles not only provides potent antibacterial activity but also confers multifunctionality,^[^ ^84^ ^]^ including high electrical conductivity of 568 S m^−1^ and reduced peak smoke rate, making these composites ideal for sterile building materials and active packaging.^[^ ^85^ ^]^

For EMI shielding, several pathways are being explored, primarily focusing on the incorporation of conductive nanofillers or the design of multifunctional layered structures. One strategy involves the in situ coating of nanomaterials onto bamboo fibers; for example, a continuous steam explosion technique to coat graphene yielded a composite with an exceptional EMI shielding effectiveness (SE) of 51.5 dB and high conductivity of 101.9 S m^−1^ at a low percolation threshold.^[^ ^86^ ^]^ Another approach utilizes functional coatings on bamboo waste powders, where incorporating Ti_3_C_2_T_x_ (MXene) nanosheets achieved an impressive EMI SE of ≈40.9 dB alongside an 89.8% increase in tensile strength.^[^ ^87^ ^]^ Alternatively, multilayered hybrid structures offer multifunctionality. A wood‐bamboo composite with an integrated copper nanoparticle layer provides an EMI SE of 40 dB plus flame retardancy,^[^ ^88^ ^]^ while a device assembling translucent bamboo with a shielding film achieves 46.3 dB SE while preserving high optical transmittance.

Despite these promising advancements, fundamental challenges must be addressed. A primary hurdle is the limited permeability of the bamboo scaffold, which often hinders the uniform penetration of functional agents and leads to inconsistent properties. While current strategies like delignification can enhance permeability, the harsh chemical processes involved generate substantial wastewater, creating a dilemma between performance and sustainability. Furthermore, for composites functionalized via surface modification, the interfacial adhesion and long‐term durability of the grown nanoparticles are paramount concerns. The stability of these functional layers against mechanical abrasion and leaching is crucial for practical application. Therefore, future progress hinges on developing greener methods to improve bamboo's permeability and engineering robust, durable interfaces for long‐lasting, high‐performance materials.

#### 3.2.2 Transparent Bamboo‐Based Materials

**Figure 6:**
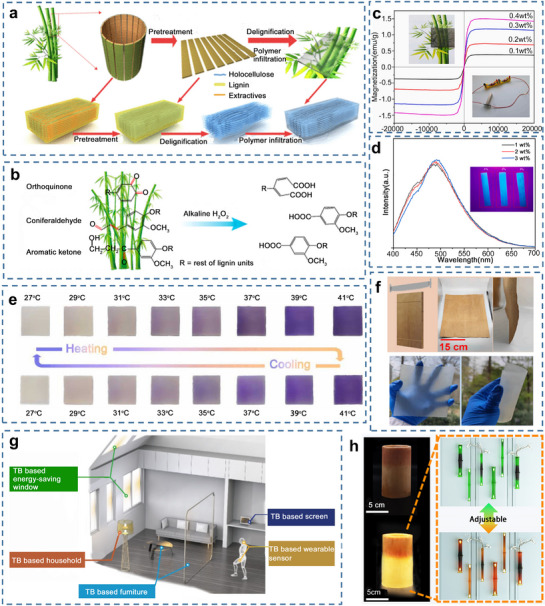
a) Preparation process diagram of TB. Reproduced with permission.^[^ ^12^ ^]^ Copyright 2020, American Chemical Society. b) Schematic diagram of lignin modification mechanism and its structural evolution. Reproduced with permission.^[^ ^93^ ^]^ Copyright 2022, Elsevier B.V. c) The magnetization curves of magnetic transparent bamboo (MTB). Reproduced with permission.^[^ ^94^ ^]^ Copyright 2022, Elsevier B.V. d) The changes of fluorescent transparent bamboo fluorescent intensity with wavelength. Reproduced with permission.^[^ ^95^ ^]^ Copyright 2022, Elsevier B.V. e) Thermochromic process of reversible thermochromic transparent bamboo (RTTB). Reproduced with permission.^[^ ^98^ ^]^ Copyright 2023, Elsevier B.V. f) The integrated application of transparent bamboo in the future. Reproduced with permission.^[^ ^90^ ^]^ Copyright 2022, Elsevier B.V. g) Physical images of the rotary‐cut bamboo veneers and TB with large‐size and flexible. Reproduced with permission.^[^ ^91^ ^]^ Copyright 2023, Elsevier B.V. h) Macroscopic view of cellulose composites (TBS) and translucent cellulose composite (TBH), and applications of TBH in lighting materials. Reproduced with permission.^[^ ^84^ ^]^ Copyright 2022, Springer Nature.

Transparent wood has long been considered a promising alternative to conventional glass materials owing to its high light transmittance, excellent thermal insulation, high impact energy absorption, and low cost.^[^ ^89^ ^]^ Since the maturity time of trees takes too long, the fast‐growing natural bamboo has attracted the research focus. Similar to the method used for preparing transparent wood, the fabrication of transparent bamboo (TB) involves the removal of lignin, a light‐absorbing substance, followed by filling with a polymer matching the refractive index of delignified bamboo templates (**Figure** **6**a,b).^[^ ^12^, ^90^, ^91^, ^92^, ^93^, ^94^, ^95^, ^96^, ^97^ ^]^ Methods for removing chromophores from wood include acidic, alkaline, and enzymatic delignification. Resin selection is a key step in resin impregnation. Polymers matching the bamboo template's refractive index include epoxy resin, polymethyl methacrylate, polylactic acid, and polyvinyl alcohol, where Figure 6a summarizes the typical preparation process for transparent bamboo (**Table** **3**).

**Table 3:** The properties and functions of TB composites.

Delignification method	Resin	Other functional additives	Transmittance	Haze	Other functions	Refs.
3 wt% NaClO_2_	Epoxy resin	–	89.60%	43.30%	–	[96]
2 wt% NaClO_2_	Epoxy resin	Thermochromic microcapsule powders	88.60%	49.83%	Reversible thermochromism, mildew resistance	[98]
1 wt% NaClO_2_	Epoxy resin	Fe_3_O_4_ nanoparticles	68.60%	49.40%	Magnetism	[94]
1% NaOH and 3 wt% NaClO_2_	Epoxy resin	Fluorescent powder (Sr_4_Al_14_O_25_:Eu^2+^, Dy^3+^)	77.30%	56.40%	Fluorescence	[95]
1% NaOH and 1 wt% NaClO_2_	Epoxy resin	1,2,3‐propanetriol‐diglycidyl‐ether	80.00%	72.00%	Large size and flexibility	[91]
3.5 wt% NaClO_2_	Epoxy resin	–	70–80%	–	–	[99]
3 wt% NaOH, 3 wt% Na_2_SiO_3_, 0.1 wt% MgSO_4_, and 4 wt% H_2_O_2_	Epoxy resin	–	87%	90%	High toughness	[93]
3.5 wt% NaClO_2_	Epoxy resin	–	78.60%	70%	Richer and more layered texture	[13]
NaClO_2_ and 30 wt% H_2_O_2_	Epoxy resin	–	89.20%	–	–	[100]
1% NaOH and 3 wt% NaClO_2_	Epoxy resin	–	80%	81%	Low thermal conductivity	[12]
4 wt% NaClO_2_	Epoxy resin	–	12%	–	–	[97]

Functional agents, such as Fe_3_O_4_ nanoparticles (Figure 6c),^[^ ^94^ ^]^ fluorescent powders (Figure 6d),^[^ ^95^ ^]^ and thermochromic powders (Figure 6e),^[^ ^98^ ^]^ can be added to the resin to achieve magnetic, fluorescent, and thermosensitive properties in TB materials. These materials have tremendous potential for engineering applications, particularly in energy‐efficient windows, light‐tunable devices, and flexible transparent materials (Figure 6f).^[^ ^90^ ^]^ Zhou and co‐workers prepared radiative cooling TB by depositing silver nanowires on delignified bamboo sheets infiltrated with epoxy resin. The long‐wavelength infrared emissivity (ɛ) for one side is as low as 0.3, to prevent the heat exchange, and the ɛ of the other side is near unity (0.95), to promote the radiative cooling. The energy‐saving simulations suggested up to 89% energy savings, when compared with commercial low‐E glass. Current TB production typically uses bamboo strips no longer than 100 mm and no wider than 50 mm. Due to bamboo's irregular, nodal, hollow, cylindrical, and highly tapered features, producing large‐sized TB is a significant challenge. The use of rotary‐cut bamboo veneers is a potential solution for achieving large TB (Figure 6g).^[^ ^91^ ^]^ Researchers also applied delignified bamboo tubes impregnated with UV resin in the fields of lighting and decorative materials, offering semi‐transparent bamboo tubes as candidates for energy‐efficient building applications (Figure 6h).^[^ ^84^ ^]^

Beyond its optical and functional properties, TB exhibits notable mechanical performance, positioning it as a promising alternative to conventional transparent materials like glass. The key to its performance lies in the synergy between the preserved cellulosic scaffold and the infiltrated polymer matrix. While the removal of lignin reduces some of the native stiffness, the polymer reinforcement endows TB with significantly higher fracture toughness and impact resistance compared to brittle glass, mitigating the risk of catastrophic failure.^[^ ^99^ ^]^ The tensile strength of TB can be tailored over a wide range, typically from 40 to 200 MPa.^[^ ^91^, ^92^, ^101^ ^]^ This variability is governed by several key factors, including the density and quality of the original bamboo, the efficacy of the delignification process, and the choice of polymer resin and impregnation completeness. For instance, Wang et al. optimized the fabrication process and reported a TB material derived from the middle layer of a bamboo culm (delignified for 5 h) that achieved a balanced performance: a tensile strength of 89.7 MPa coupled with a high light transmittance of 89.6% and a haze of 43.3%. This combination provides both structural integrity and visual privacy.^[^ ^96^ ^]^ However, different methods for producing TB exhibit distinct limitations. Chemical delignification methods, while effective, have high environmental burdens, require extended processing times, and typically employ toxic chemicals (such as NaClO and NaOH). Physical‐mechanical treatments reduce processing time but compromise microstructural integrity, resulting in decreased light transmittance and mechanical strength. Enzymatic treatments offer environmental benefits but suffer from high costs and low efficiency. All these methods face common challenges with uneven penetration when processing larger bamboo dimensions, leading to spatial heterogeneity in material properties. Furthermore, existing research predominantly focuses on enhancing optical properties while neglecting balanced optimization between optical performance, mechanical properties, and durability of transparent bamboo materials.

#### 3.2.3 Bamboo‐Based Materials for Solar Evaporators

**Figure 7:**
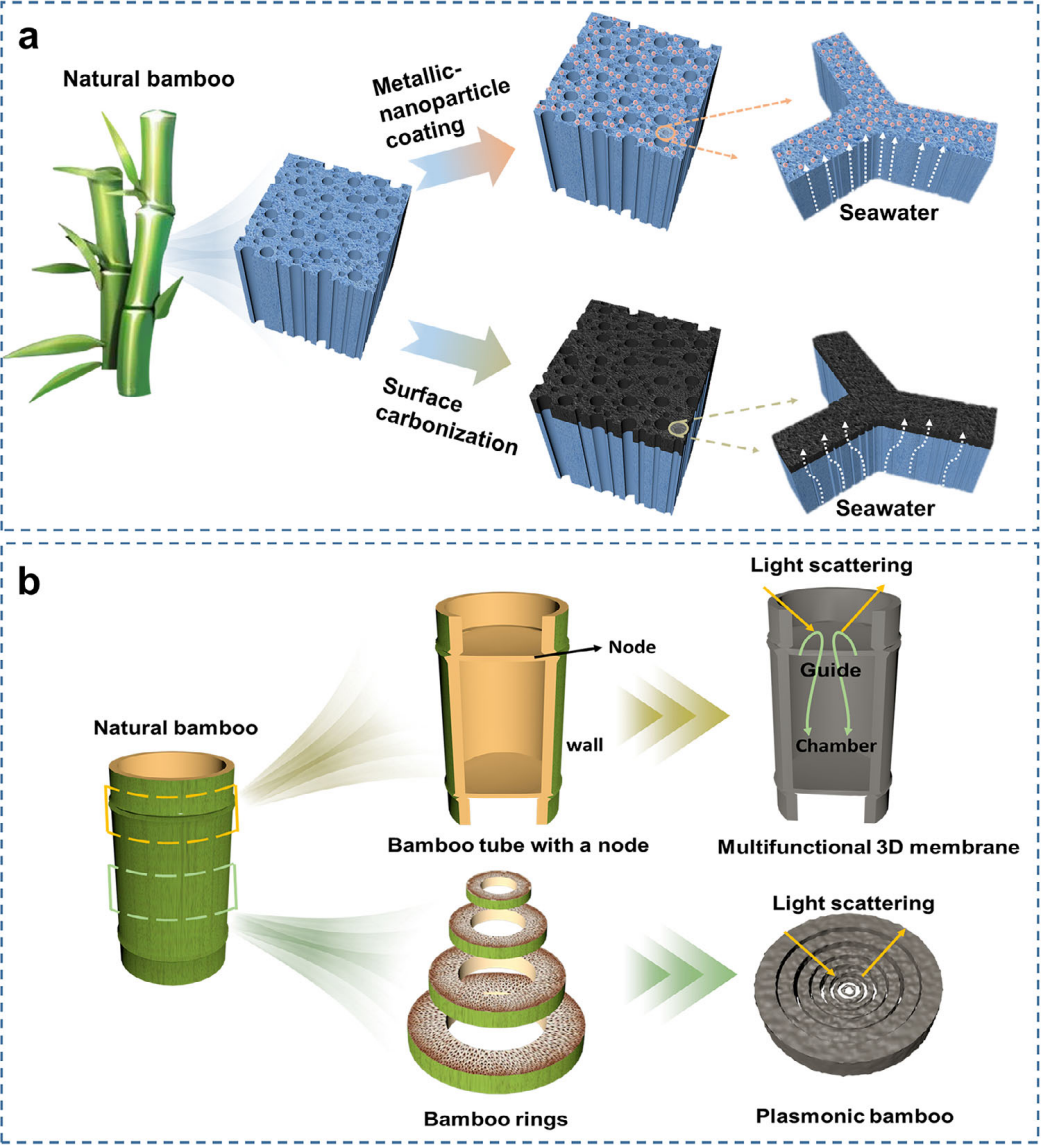
a) The implementation strategy of bamboo‐based solar evaporators. b) Two structural designs of bamboo‐based solar evaporators.

Solar evaporation is gaining attention as an effective technology to address global freshwater scarcity due to its sustainability and environmental compatibility^[^ ^102^ ^]^ (**Table** **4**). The operation of a solar evaporator involves four key steps: light absorption and conversion into heat, water transportation, evaporation, and thermal management. Bamboo has several advantageous characteristics for this. First, bamboo has oriented microchannels, commonly referred to as vessels, that efficiently convey moisture through capillary action. Second, bamboo exhibits natural hydrophilic propensity, thereby permitting water inflow into these oriented vessels. Moreover, bamboo has low thermal conductivity within these vessels, promoting heat accumulation. Consequently, natural bamboo effectively curtails heat conduction losses without compromising the water supply. However, a critical challenge arises in leveraging bamboo for solar evaporators: the need to induce robust light absorption on its surface, which is vital for concentrating solar energy to generate localized heat for vapor production. Presently, this challenge is predominantly addressed using two principal strategies: surface deposition and high‐temperature carbonization (**Figure** **7**a).

**Table 4:** An overview of current methods, structures, and performance metrics pertaining to bamboo‐based solar evaporators.

Structural modification methods	Structural design	Evaporation rate [kg m^−2^ h^−1^]	Conversion efficiency	Refs.
Surface metal nanoparticles deposition	nested bamboo ring	1.28	87%	[102]
Surface tannin acid/Fe^3+^ particles loading	bamboo strips	1.15	79%	[103]
Surface polypyrrole loading	bamboo strips	1.13	77%	[104]
Surface carbonization	cut surface in the bamboo nodes	1.65	94%	[14]
Surface carbonization	bamboo strips	3.09	–	[105]
Carbonization	bamboo strips	2.03	132%	[106]
Carbonization	cut surface in the bamboo nodes	0.96	67%	[107]

Surface deposition employs light‐absorbing materials, including metal nanoparticles,^[^ ^102^ ^]^ metal‐polyphenol networks,^[^ ^103^ ^]^ and thermally conductive polymers,^[^ ^104^ ^]^ among other options. On the other hand, carbonization can be categorized into surface^[^ ^14^, ^105^ ^]^ and bulk carbonization.^[^ ^106^, ^107^ ^]^ Surface carbonization is the most accessible and cost‐effective method. Nevertheless, owing to the constraints imposed by the thin‐walled hollow structure of bamboo, which limits the area available for photothermal conversion, the design of the device structure is particularly important (Figure 7b). One approach entails the assembly of bamboo rings with varying diameters into a nested configuration, whereas another involves segmenting bamboo into sections and utilizing the diaphragms within the bamboo nodes as the surface for photothermal conversion. The concave structures of these diaphragms effectively concentrate heat and enhance water transport, thereby increasing steam production. Remarkably, at an irradiance level of 1 kW m^−2^, evaporators featuring concave evaporating surfaces exhibit a 13.46% higher solar conversion efficiency than that of their counterparts lacking such structures. Nevertheless, the innate structure of bamboo is relatively dense, and its pore structure falls short of the development found in other porous materials. Despite achieving an evaporation rate of 3.09 kg m^−2^ h^−1^ in bamboo‐based evaporators, a substantial gap remains when compared to the reported rate of 6.10 kg m^−2^ h^−1^ for λ‐Ti_3_O_5_ deposited on polyvinyl alcohol (PVA) hydrogel.^[^ ^108^ ^]^ Furthermore, even using nested bamboo rings or node diaphragms as surfaces, the surface area of bamboo‐based solar evaporators remains limited. Therefore, enhancing the bamboo pore structure to improve fluid transport rates and developing bamboo‐based evaporators through structural designs are focal points for future research.

#### 3.2.4 Catalytic Reactors Materials

**Figure 8:**
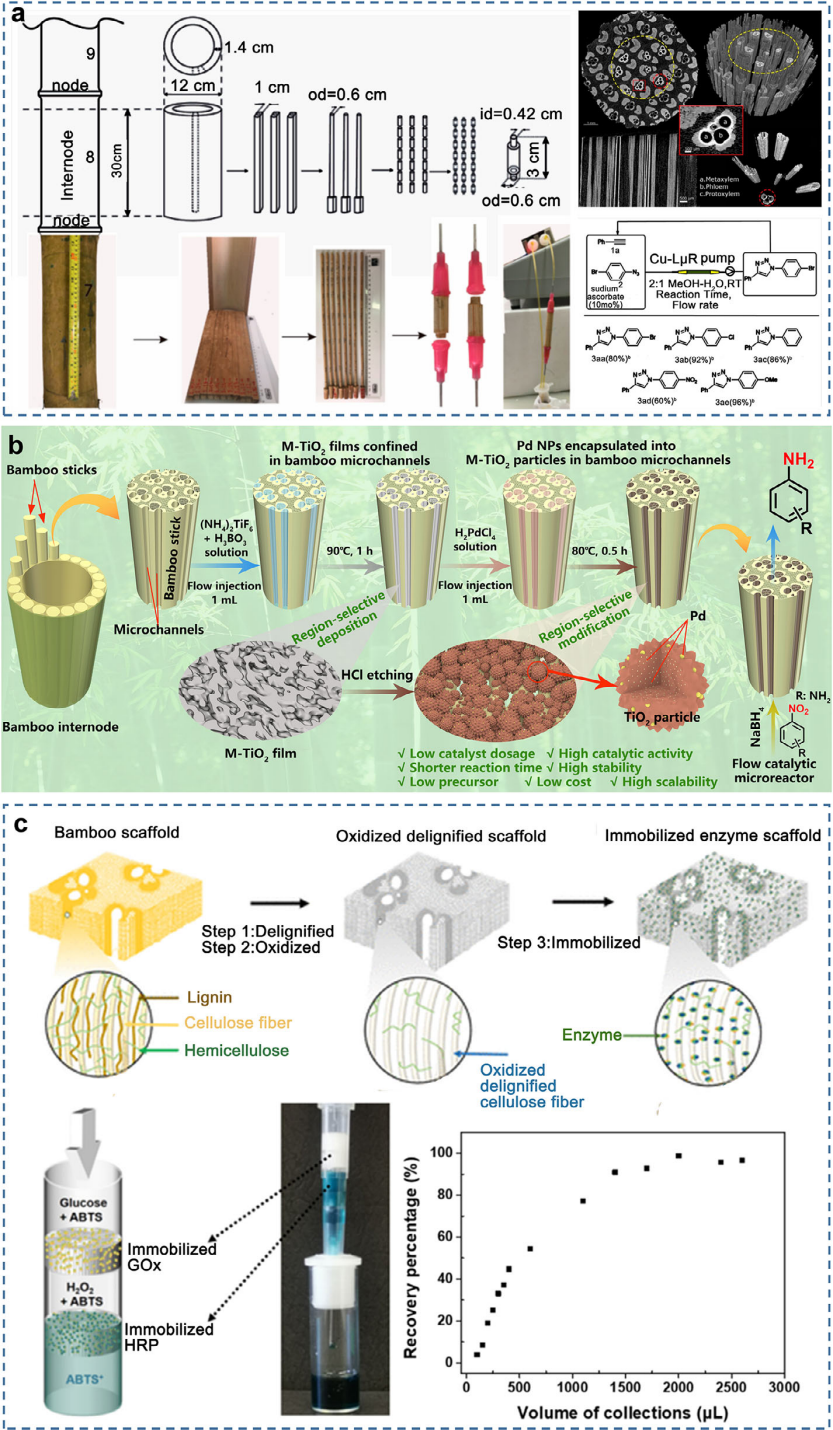
a) Schematic diagram and flow procedure scope of the lignocellulose‐based microreactor prepared with bamboo internodes, transversal and longitudinal cross‐sections of bamboo culm and three‐dimensional image with and without the vegetal biomass. Reproduced with permission.^[^ ^109^ ^]^ Copyright 2019, American Chemical Society. b) Schematic of the principle of preparing the catalytic microreactor. Reproduced with permission.^[^ ^110^ ^]^ Copyright 2023, Wiley‐VCH. c) Immobilization of the enzyme on cellulose supports, schematic diagram, physical image and recovery curves of the bioreactor. Reproduced with permission.^[^ ^111^ ^]^ Copyright 2022, American Chemical Society.

The rapid mass transfer and high‐permeability hierarchical structure of bamboo can also be used to prepare catalytic reactors based on their porosity. Bamboo can be used to fabricate microreactors with excellent catalytic activity and stability for chemical reactions. For example, it can be employed to reduce silver ammonia solutions and copper particles in situ through electrostatic interactions (**Figure** **8**a), resulting in the formation of silver nanoparticles and copper.^[^ ^109^ ^]^ Similarly, a two‐step synthesis strategy was used to prepare mesoporous TiO_2_‐encapsulated ultrafine Pd nanoparticles,^[^ ^110^ ^]^ which are confined in bamboo microchannels, forming a bamboo‐based microreactor with enhanced catalytic activity (Figure 8b). This reactor can serve as a reductant and carrier for chemical and pharmaceutical applications.

In particular, delignified bamboo exhibits increased multiscale porosity and abundant hydroxyl groups, providing sufficient sites and space for biocatalytic reactions. It serves as an ideal carrier for enzyme immobilization and the construction of hierarchical bioreactors (Figure 8c).^[^ ^111^ ^]^ Approximately 8 mg m^−2^ g^−1^ of protein can be immobilized on delignified bamboo, demonstrating excellent reusability for up to 13 cycles and high stability when stored at 4 °C for over seven weeks.

As in the case of solar evaporators, the undeveloped pore structure of bamboo limits the catalytic efficiency of bamboo‐based microreactors. Furthermore, bamboo‐based microreactors face challenges associated with the intrinsic stability of bamboo, potentially leading to the loss of bamboo components over prolonged operation.

#### 3.2.5 Bamboo‐Derived Biomass Carbon

Compared to other plants, bamboo residues exhibit higher particle density and porosity (longitudinal pores) because of their VBs and high cellulose content. Furthermore, owing to their high lignin and low ash contents, bamboo residues are suitable for producing carbon materials with high fixed carbon yields, including activated carbon (AC) and biochar^[^ ^112^, ^113^, ^114^, ^115^, ^116^, ^117^, ^118^ ^]^ (**Table** **5**). Chemical activation is commonly used to produce bamboo‐based activated carbon, with H_3_PO_4_ and KOH being the most employed reagents. Under suitable activation conditions, ACs produced using these agents can exhibit specific surface areas exceeding 2000 m^2^ g^−1^.^[^ ^119^ ^]^ These bamboo‐based ACs are used as adsorbents to remove inorganic, organic, and gaseous pollutants. Bamboo‐based ACs activated with KOH exhibit a 3D hierarchical porous structure, as well as a high surface area and high oxygen doping,^[^ ^120^ ^]^ demonstrating a remarkable adsorption capacity for Rhodamine B (>1200 mg g^−1^), thus surpassing other reported bio‐based activated carbons.^[^ ^121^ ^]^

**Table 5:** Properties and applications of carbon materials derived from bamboo material.

Carbon material type	Raw material shape	Specific surface area	Application field	Refs.
Activated carbon	Bamboo powder	2471 m^2^ g^−1^	Acid Dyes adsorption	[119]
	Bamboo powder	2133 m^2^ g^−1^	Rhodamine B adsorption	[121]
	Granular bamboo charcoals	758 m^2^ g^−1^	Yellow 161 dye adsorption	[116]
	Granular bamboo charcoals	3155 m^2^ g^−1^	Hydrogen storage	[117]
	Bamboo powder	811 m^2^ g^−1^	Carbon dioxide adsorption	[112]
	Bamboo powder	1789 m^2^ g^−1^	Flexible supercapacitor	[131]
	Bamboo powder	1988 m^2^ g^−1^	Supercapacitor	[132]
	Bamboo thin flakes	1317 m^2^ g^−1^	Supercapacitor	[133]
	Bamboo powder	745 m^2^ g^−1^	Zinc‐air battery	[120]
	Bamboo powder	226 m^2^ g^−1^	Catalytic esterification of oleic acid	[114]
Biochar/Carbonized bamboo	Bamboo culm	9.2 m^2^ g^−1^	Atrazine adsorption	[145]
	Bamboo culm	28.4 m^2^ g^−1^	Methylene blue adsorption	[146]
	Bamboo culm	70 m^2^ g^−1^	Arsenic (V) adsorption	[141]
	Bamboo chips	350 m^2^ g^−1^	Soil K increase and water holding	[140]
	Bamboo powder	629 m^2^ g^−1^	Uranium adsorption	[113]
	Bamboo chopsticks	302 m^2^ g^−1^	Gas sensors	[115]
	Bamboo sticks	–	Microfluidic self‐heating system	[144]

Bamboo‐based ACs also exhibit outstanding metal ion capture capabilities. Bamboo‐based ACs, prepared using guanidine phosphate as an activator, were rich in nitrogen and phosphorus functional groups, endowing them with high adsorption capacities for Pb(II), Cu(II), and Cd (II) in aqueous solutions.^[^ ^122^ ^]^ The adsorption capacity of bamboo‐based ACs for metal ions can be tailored not only by different bamboo species, and activators (H_2_O, CO_2_, H_3_PO_4_, etc.),^[^ ^123^ ^]^ but also by chemical modification.^[^ ^124^ ^]^ Taking bamboo‐based ACs prepared from steam‐activated Moso and Ma bamboo powders as an example, their respective adsorption capacities and removal efficiencies for heavy metal ions follow this sequence: twice‐activated Ma bamboo ACs > once‐activated Ma bamboo ACs > twice‐activated Moso bamboo ACs > once‐activated Moso bamboo ACs.^[^ ^125^ ^]^ The removal efficiency of heavy metal ions by the various bamboo ACs decreased in the order: Pb (II) > Cu (II) > Cr (III) > Cd (II). Additionally, a cation‐functionalized super adsorbent with −N^+^H_2_R groups can achieve a remarkable Cr (VI) removal capacity of 424.09 mg g^−1^.^[^ ^124^ ^]^

Bamboo‐based ACs, is also capable of generating negative ions and long‐wave infrared radiation. These negative ions effectively bond with minute dust particles, inducing their enlargement and subsequent settling to the ground. The conversion of bamboo‐based ACs into filtering materials involves composite preparations with other substances, yielding aerogels, fabrics, and membrane‐based filtering materials suitable for applications such as air filtration, wastewater treatment, and bio‐purification. An illustrative example in air filtration encompasses the development of a composite network structure comprising silver nanowires and bamboo ACs on a nylon sheet, resulting in a filter with efficient PM2.5 removal.^[^ ^126^ ^]^ Utilizing chitosan (CS) as the skeletal matrix, bamboo ACs as functional particles, and methyltrimethoxysilane (MTMS) as a hydrophobic modifier introduced into the CS system, a composite aerogel obtained through freeze‐drying exhibited outstanding removal capabilities for both PM2.5 (filtration rate of 94.2%) and formaldehyde (adsorption capacity of 61.6 mg g^−1^).^[^ ^127^ ^]^ Another instance involves an aerogel air filter composed of cellulose nanofibrils (CNF), PVA, and bamboo activated charcoal, achieving a remarkable 95% efficiency in PM2.5 filtration.^[^ ^128^ ^]^ For wastewater treatment, nanoscale AC powder incorporated into polyester fibers effectively enhances dissolved oxygen levels while mitigating biochemical oxygen demand.^[^ ^129^ ^]^ For bio‐purification, a polyurethane foam amalgamated with polyurethane and bamboo ACs attained a maximum removal of 12.68 g m^−3^ h^−1^ for *n*‐hexane and surpassed 30.28 g m^−3^ h^−1^ for dichloromethane degradation.^[^ ^130^ ^]^

**Figure 9:**
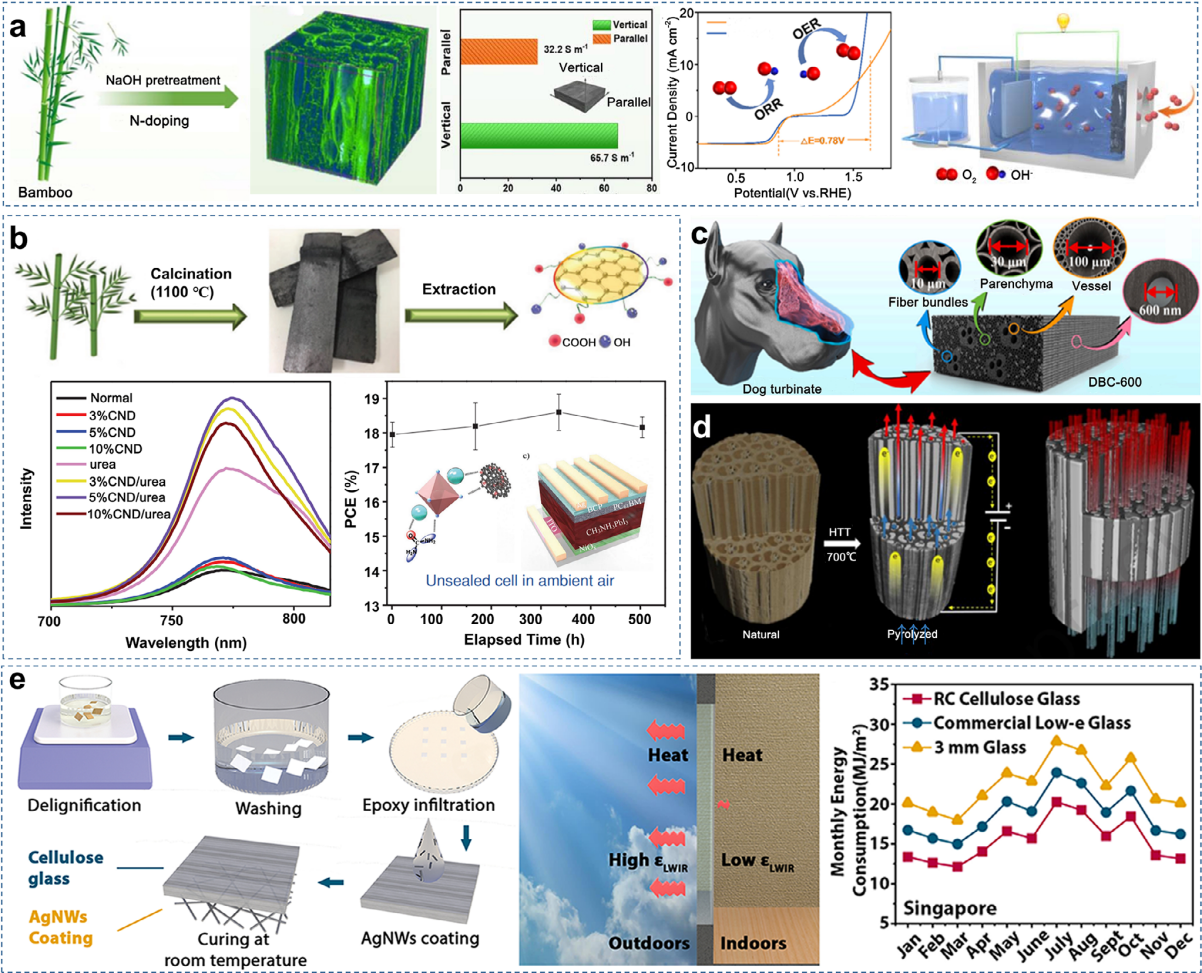
a) Microscopic 3D structure, electrical conductivity and oxygen evolution reaction polarization curves of N‐doped pre‐treated bamboo‐based hierarchical porous carbon (NPBC), and schematic illustration of a liquid flow zinc‐air batteries with NPBC. Reproduced with permission.^[^ ^120^ ^]^ Copyright 2023, Royal Society of Chemistry. b) Preparation of carbon nanodots (CNDs) from carboned bamboo powder, photoluminescence spectra of CNDs, and power conversion efficiencies and structure of perovskite solar cell devices incorporating carbon‐based additives. Reproduced with permission.^[^ ^142^, ^143^ ^]^ Copyright 2018, Wiley‐VCH. c) Tubular structure of carbon materials derived from disposable bamboo chopsticks similar to that of dog turbinate. Reproduced with permission.^[^ ^178^ ^]^ Copyright 2023, Elsevier B.V. d) Schematic of the preparation process and fluid warming of the bamboo‐based microfluidic heater. Reproduced with permission.^[^ ^115^ ^]^ Copyright 2023, Elsevier B.V. e) Schematic manufacturing procedures, working mechanism and simulated monthly energy consumption of the radiative cooling cellulose glass. Reproduced with permission.^[^ ^149^ ^]^ Copyright 2021, American Chemical Society.

Bamboo‐based AC also serves as an active electrode material in flexible supercapacitors and batteries (**Figure** **9**a,b).^[^ ^120^, ^131^, ^132^, ^133^, ^134^ ^]^ Recently, its application has been extended to the burgeoning field of sodium‐ion batteries. The inherent hierarchical porous structure of bamboo‐derived carbon provides abundant active sites for Na^+^ storage and facilitates rapid ion diffusion, leading to high capacity and excellent rate performance.^[^ ^135^, ^136^, ^137^, ^138^ ^]^ A notable strategy involves using deep eutectic solvents to treat bamboo, followed by carbonization to produce hard carbon anode materials. This approach yields a material with a unique closed‐pore structure that enables exceptional electrochemical performance, even without conductive additives. For instance, such a hard carbon anode has demonstrated an impressive reversible capacity of 348.6 mAh g^−1^ at 0.1 C, outstanding rate capability with 201.4 mAh g^−1^ retained at a high rate of 10 C, and exceptional cycling stability, maintaining 90% of its capacity after 300 cycles at 1  C.^[^ ^139^ ^]^ A zinc‐air battery using an N‐doped bamboo‐based AC catalyst as the air cathode exhibited a peak power density of 249 mW cm^−2^ and a long‐term stability of 300 h. These performance metrics highlight the significant potential of engineered bamboo‐derived carbons as sustainable, high‐performance anodes for next‐generation energy storage. Moreover, bamboo‐derived biochar, obtained through bamboo pyrolysis, has emerged as an effective alternative to bamboo‐based ACs for certain adsorption applications. It has the added advantage of low production costs owing to the absence of high‐temperature activation steps.^[^ ^140^ ^]^ Although its specific surface area may only reach several hundred m^2^ g^−1^, chemical modification with agents such as acids, alkalis, and nanoparticles can enhance the porosity and surface characteristics of bamboo‐derived biochar. For instance, the deposition and dispersion of Fe_3_O_4_ nanoparticles (10–25 nm) and their aggregates on the biochar surface increased the surface area four‐fold to ≈70 m^2^ g^−1^.^[^ ^141^ ^]^

Furthermore, bamboo materials can be directly carbonized in the form of strips or blocks. However, the overall carbonization of bamboo leverages the fluid channels within bamboo wood to a greater extent. Discarded disposable bamboo chopsticks have also been carbonized to produce carbon materials that exhibit structural similarities to dog‐nose beetles (Figure 9c). These materials have tubular structures and can be employed in NH_3_ gas sensors. After 50 days, the sensor exhibited a response fluctuation of less than 4.2% to NH_3_, with excellent recovery capability.^[^ ^142^, ^143^ ^]^ Such tubular structures can be used as 3D microfluidic heaters for heating polar solvents (H_2_O and ethylene glycol, Figure 9d).^[^ ^144^ ^]^

Current bamboo‐based carbon materials research faces several critical limitations: First, most activation methods (using KOH, H_3_PO_4_, etc.) have high environmental burdens, with chemical waste treatment costs constraining industrial‐scale production. Second, precise control of pore structure remains challenging, resulting in poor reproducibility of material performance. Third, conventional pyrolysis carbonization processes are energy‐intensive, contradicting low‐carbon production principles. Finally, inconsistent characterization methods across different studies make performance comparisons difficult. Moreover, while bamboo‐based carbon materials demonstrate excellent performance in laboratory settings, maintaining performance consistency and cost control during scale‐up from laboratory to industrial scale remains an urgent challenge requiring systematic investigation.

#### 3.2.6 Bamboo‐Based Materials for Thermal Management

Due to its hierarchical porous structure and biopolymer composition, bamboo demonstrates excellent thermal management properties, which makes it suitable for thermally insulated roofs^[^ ^147^ ^]^ and cooling‐tower fillers. Two primary directions have been pursued for the thermal management of bamboo. One approach is to reduce the thermal conductivity of bamboo to enhance its insulating properties. These methods increase the abundance of nanoscale pores within cell walls, disrupting phonon transport and eliminating thermally conductive lignin. This leads to an even lower thermal conductivity of cellulose nanofibers (0.1 × 10^−6^ K^−1^).^[^ ^78^ ^]^ Cellulose can also serve as a photonic solar converter and thermal emitter, making it a promising candidate for cooling materials during daytime radiative cooling achieved through the densification of delignified bamboo.^[^ ^148^ ^]^ For example, a bamboo cellulose glass is obtained by delignification of natural bamboo slices following with epoxy infiltration and silver nanowires coating, which has high optical transparency and significantly enhanced radiative cooling. According to the energy saving simulations, the radiative cooling cellulose glass outperforms the commercial low emissivity glass, up to 89% annually^[^ ^149^ ^]^ (Figure 9e). The other approach focuses on enhancing the thermal conductivity of bamboo. It employs the methods introduced earlier in Section 3.2.3 for solar evaporators, including processes such as high‐temperature carbonization or incorporation of thermally conductive materials. For example, coating bamboo with graphene and cellulose nanocrystals can substantially increase the thermal conductivity of bamboo fiber fabric to 0.136 W m^−1^ K^−1^. This indicates a significant potential of bamboo fiber for the development of cooling textiles.^[^ ^150^ ^]^ Given bamboo's natural insulation properties, future advancements may lead to cost‐effective structural modifications for developing thermally conductive materials from bamboo.

### 3.3 Utilization of Bamboo Cells to Fabricate Materials

Bamboo is composed of elongated fiber cells and square‐shaped parenchyma cells (PCs), which provide mechanical support and facilitate the transport of water and inorganic salts. The PCs form a foam‐like structure, enveloping the BFs to protect their mechanical toughness while also providing storage space and transportation pathways for nutrients. As the separation methods for these two types of cells are being developed, research has increasingly focused on bamboo design at the cellular level based on structural and performance characteristics. It should be specifically emphasized that the application section for fiber cells encompasses not only the utilization of single fibers but also macrofiber applications. BFs primarily serve as reinforcement materials for high‐strength composites, while individual fibers are better suited for precision applications.

#### 3.3.1 Fiber Cells

**Figure 10:**
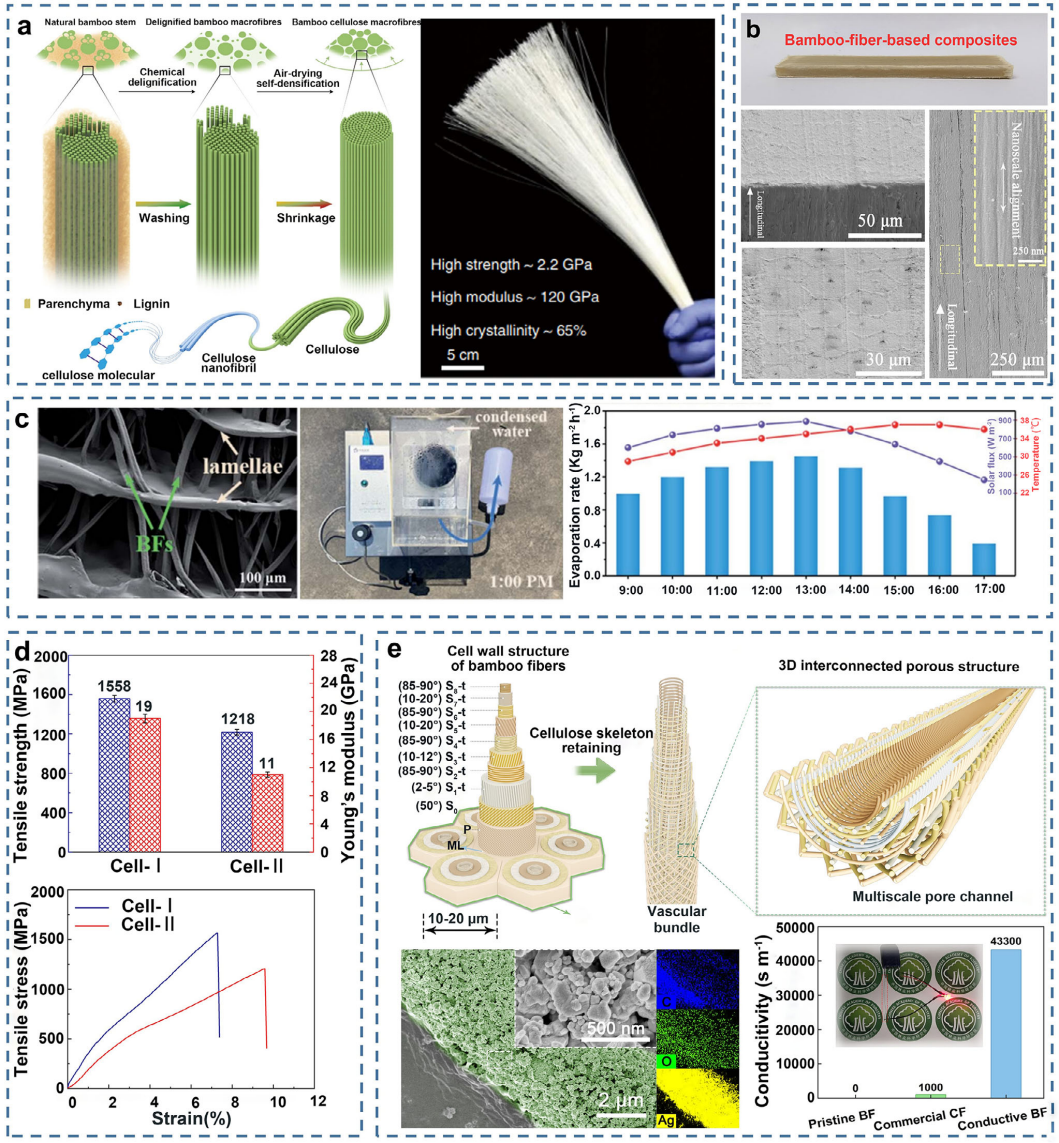
a) Schematic diagram and physical image of high‐performance cellulose macrofibres extracted from natural bamboo. Reproduced with permission.^[^ ^155^ ^]^ Copyright 2022, Springer Nature. b) SEM images of bamboo‐fiber‐composites at different views and magnifications. Reproduced with permission.^[^ ^158^ ^]^ Copyright 2022, Elsevier B.V. c) SEM image of chitosan/bamboo fibers, and physical image, synchronous solar intensity, ambient temperature, and water evaporation rates of sponge evaporator from BFs. Reproduced with permission.^[^ ^159^ ^]^ Copyright 2021, Royal Society of Chemistry. d) Tensile strength and tensile stress–strain curves of cellulose‐I and cellulose‐II. Reproduced with permission.^[^ ^158^ ^]^ Copyright 2022, Elsevier B.V. e) Schematic diagram of the bamboo fiber cell wall structure and the hollow 3D porous structure of the cellulose skeleton, and SEM images, C, O, Ag element maps and conductivity of Ag‐coated BFs. Reproduced with permission.^[^ ^18^ ^]^ Copyright 2022, Springer Nature.

BFs are the primary source of mechanical strength in bamboo. The secondary walls of fiber cells consist of cellulose microfibers and filaments oriented in distinct directions in the thin and thick layers. Specifically, the thick layers feature a near‐parallel orientation to the fiber axis, providing high tensile strength and stiffness, while the thin layers have a larger helical angle, contributing to toughness and interfacial shear strength. This multi‐layered, helicoidal structural arrangement imparts the fibers with superior and stable mechanical properties, forming the fundamental basis of the exceptional macroscopic mechanical performance of bamboo.^[^ ^151^ ^]^ Macrofibers, consisting of many single fibers, can be extracted from bamboo culms by physical, chemical, or biological methods.^[^ ^152^, ^153^, ^154^ ^]^ Macrofibers extracted through a mild delignification chemical process exhibit elevated tensile strength and Young's modulus, reaching remarkable values of 2.2 and 120 GPa, respectively (**Figure** **10**a). This strength‐to‐weight ratio significantly surpasses those of materials such as silk, synthetic polymers, and glass fibers.^[^ ^155^ ^]^ These extracted macrofibers are useful in plastic composite materials, cement mortars, and concrete to enhance the material mechanical properties and restrict crack propagation within the matrix (Figure 10b).^[^ ^156^, ^157^, ^158^ ^]^ Besides reinforcing, when dispersed in polymers, macrofiber help form interconnected macroporous structures, interacting with chitosan for efficient and durable sponge evaporator production (Figure 10c).^[^ ^159^ ^]^

It is important to note that the properties of these extracted fibers can be precisely tailored by controlling their crystalline structure. The native fibers are composed of cellulose I, which features a parallel‐chain crystal packing and an extensive hydrogen‐bonding network that endows it with high intrinsic strength and stiffness. However, by manipulating processing conditions, such as the concentration of the alkali solution during treatment, a solid‐state transformation from cellulose I to cellulose II can be induced. Cellulose II possesses an antiparallel chain arrangement and a distinct hydrogen‐bonding network. While this transformation results in fibers that are ≈22% lower in strength than their cellulose I counterparts, it significantly enhances their flexibility and ductility. This trade‐off makes cellulose II fibers highly suitable for creating flexible materials, such as substrates for wearable electronics and advanced textiles (Figure 10d).^[^ ^158^ ^]^ Interestingly, chemically extracted pure cellulose fiber retain a three‐dimensional cellulose skeleton‐based porous structure owing to the removal of lignin. This structure can accommodate various functional nanoparticles (such as silver, copper, nickel, and antimony‐doped tin oxide)^[^ ^160^, ^161^ ^]^ and polymers (polyphenols and organic metal frameworks).^[^ ^162^ ^]^ Single BFs loaded with nanosilver exhibit an impressive electrical conductivity of up to 43 300 S m^−1^ and a tensile strength of 760 MPa, making them ideal electrode materials for flexible capacitors^[^ ^18^ ^]^ (Figure 10e). Fabrics made from prepared BFs, with their unique micro/nano ridge/groove structures, generate capillary forces at multiple scales, leading to strong self‐cleaning properties.^[^ ^163^ ^]^ Moreover, cross‐scale synergistic applications stand as an innovative strategy. The synthesis of multiscale bamboo cellulose‐based materials involves multiscale fiber extraction, surface modification, and ion cross‐linking for assembly, yielding a sustainable material with moldability, biocompatibility, biodegradability, and excellent mechanical properties for plastic alternatives.^[^ ^164^ ^]^

#### 3.3.2 Parenchyma Cells

**Figure 11:**
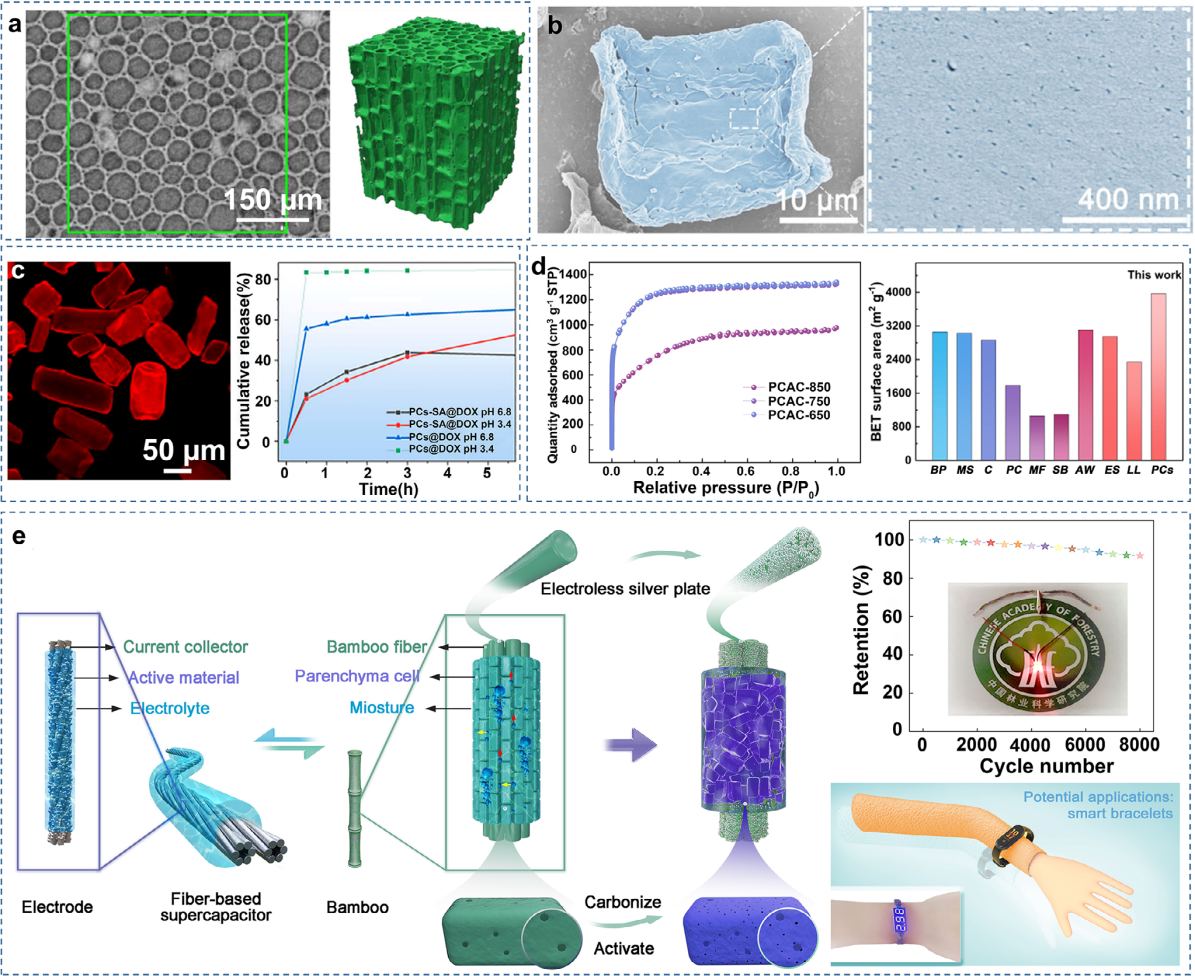
a) Visualization of the parenchyma tissue by micro‐CT and 3D reconstruction. Reproduced with permission.^[^ ^166^ ^]^ Copyright 2018, Elsevier B.V. b) Microstructure of KOH‐activated PC. Reproduced with permission.^[^ ^18^ ^]^ Copyright 2022, Springer Nature. c) SEM images of PCs, drug loading and hollow lignocellulose microcapsules after doxorubicin(dox) release, and cumulative release PCs with dox in different pH media. Reproduced with permission.^[^ ^168^ ^]^ Copyright 2022, American Chemical Society. d) N_2_ adsorption–desorption curves of different activation temperature activated carbon from PCs and BET statistics of different biomass activated carbon. Reproduced with permission.^[^ ^170^ ^]^ Copyright 2023, Elsevier B.V. e) Fabrication process, cycle performance and application of the flexible bamboo‐structured supercapacitors. Reproduced with permission.^[^ ^18^ ^]^ Copyright 2022, Springer Nature.

Despite constituting over 50% of the bamboo tissue volume, PCs have historically been considered a low‐value byproduct in processes like pulping.^[^ ^165^ ^]^ However, their unique structural attributes present significant opportunities for advanced applications. PCs are characterized by their hollow lumens, multi‐layered thin walls, and a highly porous structure (**Figure** **11**a,b).^[^ ^18^, ^166^, ^167^ ^]^ Structurally, PCs have a much lower density (typically 0.3–0.5 g cm^−^^3^) and higher porosity compared to BFs. Their chemical composition is also distinct, featuring a lower cellulose content (≈30–40%), higher proportions of hemicellulose, and a significant amount of non‐structural components. These components, primarily starch, proteins, and other extractives, are often referred to as “impurities” in the context of pulp and paper manufacturing as they can interfere with processing and reduce yield. Their cell walls are thinner and less organized, resulting in poor mechanical properties (low strength and stiffness) but high compressibility, which allows them to function as a cushioning matrix in the natural bamboo structure. Mechanically, the thin, less‐organized cell walls of PCs result in poor tensile and flexural properties. For instance, the tensile strength of individual PC tissue is typically below 30 MPa, an order of magnitude lower than that of fiber bundles. However, this translates to high compressibility and low stiffness, allowing PCs to function effectively as a cushioning and energy‐absorbing matrix in the native bamboo structure. This unique combination of high porosity and compressibility makes PCs an intriguing raw material for applications such as lightweight fillers, porous carbon precursors, and micro‐encapsulation carriers. By leveraging the natural capsule structure of PCs and their abundant surface oxygen functionalities, they can be processed into degradable microcapsules with a high encapsulation efficiency of 92.2% for doxorubicin hydrochloride (Figure 11c).^[^ ^168^ ^]^ Carbonized bamboo parenchymal cells retain their hollow internal structure, accommodating phase‐change materials like pure polyethylene glycol, leading to a 128% increase in thermal conductivity and enhanced phase‐change energy storage capacity.^[^ ^169^ ^]^

The highly porous structure and loose multilayered configuration of the PCs facilitate the impregnation of activators and create more activation sites.^[^ ^171^ ^]^ This enables the preparation of hierarchical porous activated carbon with a high specific surface area of up to 4000 m^2^ g^−1^ (Figure 11d), which far exceeds those of other biomass precursor materials for activated carbon.^[^ ^170^ ^]^ This hierarchical porous activated carbon serves as an efficient adsorbent for heavy metal removal^[^ ^172^ ^]^ and as an active material for energy storage applications when used as an electrode material. By combining the activated thin‐walled PCs with silver‐loaded conductive BFs, they can be reconfigured into bamboo‐structured flexible supercapacitors. At a surface power density of 119 µW cm^−2^, the areal energy density reaches 65 µW h cm^−2^. This lightweight, cost‐effective, and high‐energy‐density bamboo‐structured flexible supercapacitor has the potential for numerous intelligent textile applications (Figure 11e).

Although researchers have utilized BFs and PCs at the cellular scale, the relationship between the structure and performance of individual cells remains unclear. Various separation processes and alterations in the process parameters can significantly influence the pore structure, crystal composition, and chemical composition of these cell types. Future research should focus on the regulation of the morphology and structure of these two cell types to adapt them to different application needs. Furthermore, the separation efficiency of the two cell types and the low yield of the PCs are urgent issues that need to be addressed.

### 3.4 Functional Materials Obtained from Chemical Components

Bamboo primarily comprises three chemical components: cellulose, hemicellulose, and lignin. These compounds can be transformed into various high‐performance materials and utilized in applications such as composites, energy storage, and water purification.^[^ ^173^, ^174^ ^]^

#### 3.4.1 Cellulose‐Based Functional Materials

Cellulose, the primary structural component of bamboo, is a linear polysaccharide renowned for its high crystallinity, robust mechanical strength, and versatile chemical functionality afforded by its abundant hydroxyl groups.^[^ ^175^, ^176^ ^]^ Bamboo cellulose and wood‐derived cellulose are structurally very similar, both consisting of β‑1→4‐linked glucose chains and typically exhibiting the cellulose I crystalline form. Previous studies have shown that, in certain samples or under specific extraction treatments, the average degree of polymerization of bamboo cellulose may be slightly lower than that of some wood‐derived celluloses.^[^ ^177^ ^]^ Meanwhile, its crystallinity index generally falls within a medium to high range, comparable to or even slightly higher than that of certain wood celluloses. Additionally, the microfibrils in bamboo cellulose are more readily exposed during chemical treatments, which can result in a more “accessible” crystalline structure during some processing steps. Research has extensively focused on isolating and transforming bamboo‐derived cellulose into advanced materials such as nanocellulose, regenerated fibers, aerogels, hydrogels, films, and carbon‐based nanomaterials.

**Figure 12:**
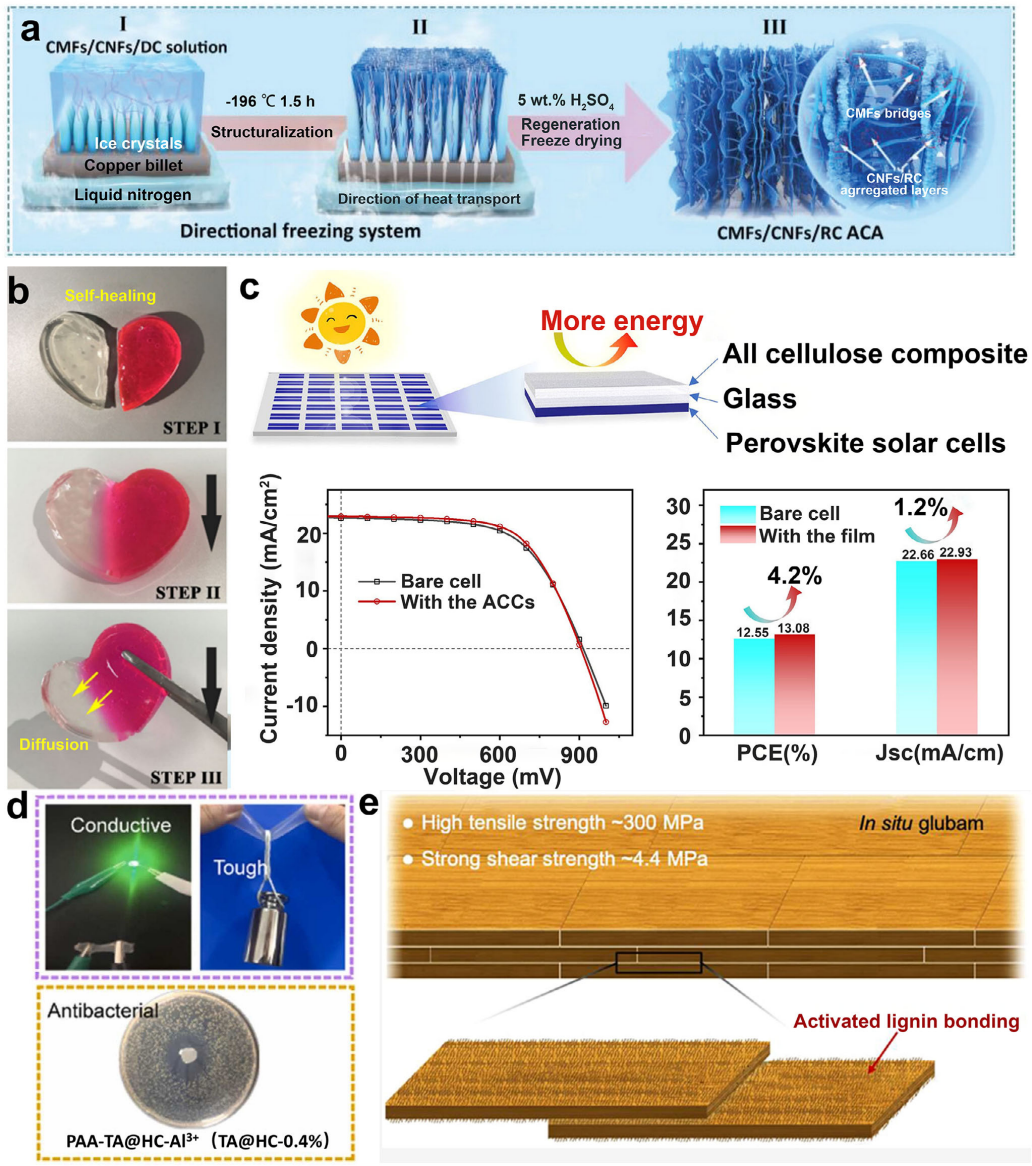
a) The strategy of constructing cellulose microfibers (CMFs)/cellulose nanofibers (CNFs)/regenerated cellulose (RC) all‐cellulose aerogel (ACA). Reproduced with permission.^[^ ^178^ ^]^ Copyright 2023, Wiley‐VCH. b) Cellulose hydrogel with excellent shape control, high sensitivity and self‐healing properties as a highly sensitive strain sensor for wearable human‐activity monitoring.^[^ ^180^ ^]^ Reproduced with permission. Copyright 2022, Elsevier B.V. c) Schematic diagram of perovskite solar cell surface enclosing all cellulose composites and their current density–voltage curves and performance comparison. Reproduced with permission.^[^ ^185^ ^]^ Copyright 2021, Elsevier B.V. d) Hemicellulose‐based hydrogel with electrical conductivity, flexibility, and antibacterial properties. Reproduced with permission.^[^ ^191^ ^]^ Copyright 2022, Elsevier B.V. e) Schematic of stacking softened bamboo into a multilayer in situ glubam board using lignin as the only in situ glue.^[^ ^193^ ^]^ Reproduced with permission. Copyright 2023, American Chemical Society.

*Aerogels*. The fabrication of bamboo cellulose aerogels typically involves disrupting the native intermolecular hydrogen bonds to dissolve the cellulose, followed by regeneration and drying (e.g., freeze or supercritical drying).^[^ ^178^ ^]^ This process creates a lightweight, highly porous, 3D nanoporous network. The resulting high specific surface area and density of surface hydroxyl groups are directly responsible for its excellent capacity to adsorb heavy metal ions, dyes, and other impurities. This functionality can be tailored; for instance, grafting hydrophobic alkyl chains onto the cellulose hydroxyls via silanization enhances its oleophilicity, making it ideal for oil spill cleanup.^[^ ^179^ ^]^ Furthermore, these aerogels serve as versatile scaffolds for creating multifunctional composites. As illustrated in **Figure** **12**a, by simply impregnating the porous cellulose network with functional additives—such as hydroxyapatite, carboxyl carbon nanotubes, or LiCl—their intrinsic properties can be enhanced. The functional groups on these additives interact with the abundant hydroxyls on the cellulose backbone, resulting in advanced composites with tailored properties for applications including thermal insulation, electromagnetic interference shielding, and piezoresistive sensing^[^ ^178^ ^]^ (Figure 12a).

*Hydrogels*. The unique structural evolution during hydrogel formation governs its properties. For example, in a bamboo cellulose/PVA double‐network hydrogel, a mechanically robust framework is formed where rigid cellulose nanofibers provide structural reinforcement, while dynamic borate‐diol ester bonds with the flexible PVA network confer self‐healing properties and high ionic conductivity, enabling its use as a strain sensor^[^ ^180^ ^]^ (Figure 12b). Furthermore, the porous network of these hydrogels serves as an excellent reservoir for entrapping or conjugating antibacterial agents, leading to materials with exceptional wound healing capabilities.^[^ ^181^, ^182^ ^]^

*Films*. Dissolving bamboo cellulose in solvents like ionic liquids, the hydrogen‐bonded chains are disentangled, allowing for regeneration into dense, non‐porous films via phase transition.^[^ ^183^ ^]^ The resulting films are typically transparent, biodegradable, and highly hydrophilic.^[^ ^184^ ^]^ A notable application is the all‐cellulose composite film, where partially dissolved cellulose acts as a molecular binder for undissolved fibers. This creates a structure with minimal interfacial defects, leading to high optical transparency (82.4–90%) and tunable haze, which has been shown to improve the power conversion efficiency of perovskite solar cells (Figure 12c).^[^ ^185^ ^]^

*Carbon‐based nanomaterials*. Carbon dots (CDs) can be synthesized from bamboo cellulose via pyrolysis or hydrothermal carbonization. This process involves a series of chemical transformations—dehydration, dehydrogenation, and aromatization—that convert the polysaccharide backbone into carbonaceous nanoparticles. These CDs inherit abundant oxygen‐containing functional groups from the cellulose precursor, which are crucial for their high stability, unique optical properties, and suitability as drug delivery carriers after further surface modification.^[^ ^186^ ^]^

#### 3.4.2 Hemicellulose‐Based Functional Materials

Hemicellulose, the second most abundant polysaccharide in bamboo, is primarily a heteropolymer composed of a xylan backbone with various side chains. A key characteristic that distinguishes bamboo hemicellulose from that of many softwoods is its dominant hemicellulose being a highly acetylated O‑acetyl‑4‑O‑methylglucurono‑xylan (often termed 4‑O‑methylglucurono‑arabino‑xylan), whereas softwoods mainly contain galactoglucomannan and smaller amounts of arabinoglucuronoxylan.^[^ ^187^ ^]^ The bamboo xylan is more amorphous in nature and does not bind as tightly to cellulose microfibrils, making it more loosely associated in the cell wall matrix. This structural feature makes it relatively easy to extract from bamboo using mild alkaline or hydrothermal treatments. Once extracted, its rich chemical functionality—including hydroxyl, acetyl, and carboxyl groups—makes it a versatile platform for creating advanced functional materials.

The chemical structure of bamboo hemicellulose is readily tailored to achieve specific functionalities.^[^ ^174^, ^188^ ^]^ For instance, hydrolysis cleaves the glycosidic bonds to produce bioactive xylooligosaccharides (XOS) and xylose.^[^ ^189^ ^]^ These smaller molecules can then be further transformed; catalytic hydrogenation of xylose reduces the aldehyde group to a hydroxyl group, yielding xylitol, a valuable sugar substitute with anti‐caries properties. Alternatively, XOS can be used directly as prebiotics or for their immune‐stimulating activity.

Hemicellulose's polymer backbone itself can be engineered into functional materials. Its abundant hydroxyl groups enable the formation of films with excellent oxygen barrier properties, suitable for food packaging. By chemically modifying the side chains and cross‐linking the polymer, self‐healing hydrogels can be fabricated. For example, nanoparticles derived from modified hemicellulose can be assembled into nanofilms for wearable strain sensors, where the high density of functional groups contributes to a high strain sensitivity (Gauge Factor =  8.34) (Figure 12d).^[^ ^190^ ^]^ Furthermore, the carboxyl and hydroxyl groups on the hemicellulose chains act as effective chelating sites for metal ions, making it a natural adsorbent for heavy metal removal.^[^ ^174^ ^]^ They can also serve as reducing and stabilizing agents for the in situ synthesis of silver nanoparticles, demonstrating its role as a functional biopolymer in nanomaterial fabrication.^[^ ^191^ ^]^

#### 3.4.3 Lignin‐Based Functional Materials

Lignin, a complex amorphous aromatic polymer, provides structural rigidity to the bamboo culm. A distinguishing feature of bamboo lignin is its H‐G‐S co‐polymeric structure,^[^ ^46^ ^]^ consisting of all three monolignol units—p‐coumaryl (H), coniferyl (G), and sinapyl (S) alcohols. Bamboo lignin typically contains significant proportions of S and G units alongside smaller amounts of H units and often exhibits acylation (e.g., p‐coumarate or acetate residues), which is characteristic of grass lignins. This composition differs from typical softwoods (mostly G‐type) and hardwoods (G‐S type) and results in a more linear, less condensed structure with a higher proportion of reactive β‐O‐4 ether linkages. This unique chemical makeup makes bamboo lignin relatively easier to depolymerize and functionalize.

The functionalization of bamboo lignin primarily leverages its abundant phenolic hydroxyl, carboxyl, and methoxy groups.^[^ ^173^ ^]^ These native functional groups are responsible for its intrinsic antioxidant, UV‐blocking,^[^ ^192^ ^]^ and antibacterial properties.^[^ ^191^ ^]^ For instance, low‐molecular‐weight lignin fractions extracted from bamboo exhibit enhanced antibacterial activity, attributed to their higher concentration of phenolic hydroxyl groups.

Moreover, lignin's structure can be strategically employed in advanced composites. In one approach, it serves as a bio‐based curing agent and toughener for epoxy resins.^[^ ^194^ ^]^ Here, the phenolic hydroxyls on the lignin backbone react with the epoxy groups, creating a cross‐linked network that improves the thermal stability and mechanical performance of the final thermoset. A more innovative strategy utilizes the in situ lignin within the bamboo scaffold as a natural adhesive^[^ ^193^ ^]^ (Figure 12e). During a partial delignification and thermocompression process, the remaining lignin softens and flows, acting as a thermoplastic glue that coats and cross‐links the cellulose nanofibers. This process creates an ultra‐strong, covalently bonded interface, yielding a dense, high‐performance composite without any external adhesive. The rich aromatic structure of lignin also makes it an excellent precursor for carbon‐based materials, such as carbon fibers and adsorbents.^[^ ^195^ ^]^

While functionalizing bamboo's primary constituents offers a promising route to high‐value products, significant challenges hinder their widespread implementation. The overarching issue is the lack of selective and efficient fractionation technologies; current processes often degrade the native biopolymers, for instance by reducing cellulose's degree of polymerization or condensing lignin, which compromises their ultimate performance. Furthermore, strategies are needed to leverage the unique structural advantages of bamboo's polymers—such as the high accessibility of its cellulose and the reactivity of its G‐S‐H lignin—without destroying these traits during conversion. Ultimately, bridging the gap between laboratory‐scale success and industrial viability requires integrated biorefinery approaches that enable clean component separation and cost‐effective conversion, paving the way for a true circular bamboo bioeconomy.

## 4 The Advancement in ML and AI for Bamboo and Bamboo‐Based Materials

Machine learning (ML) and artificial intelligence (AI) have recently gained attention as transformative technologies in the development and optimization of bamboo‐based materials, providing data‐driven frameworks to enhance prediction accuracy, improve manufacturing efficiency, and guide structure–property relationships.^[^ ^196^ ^]^

**Figure 13:**
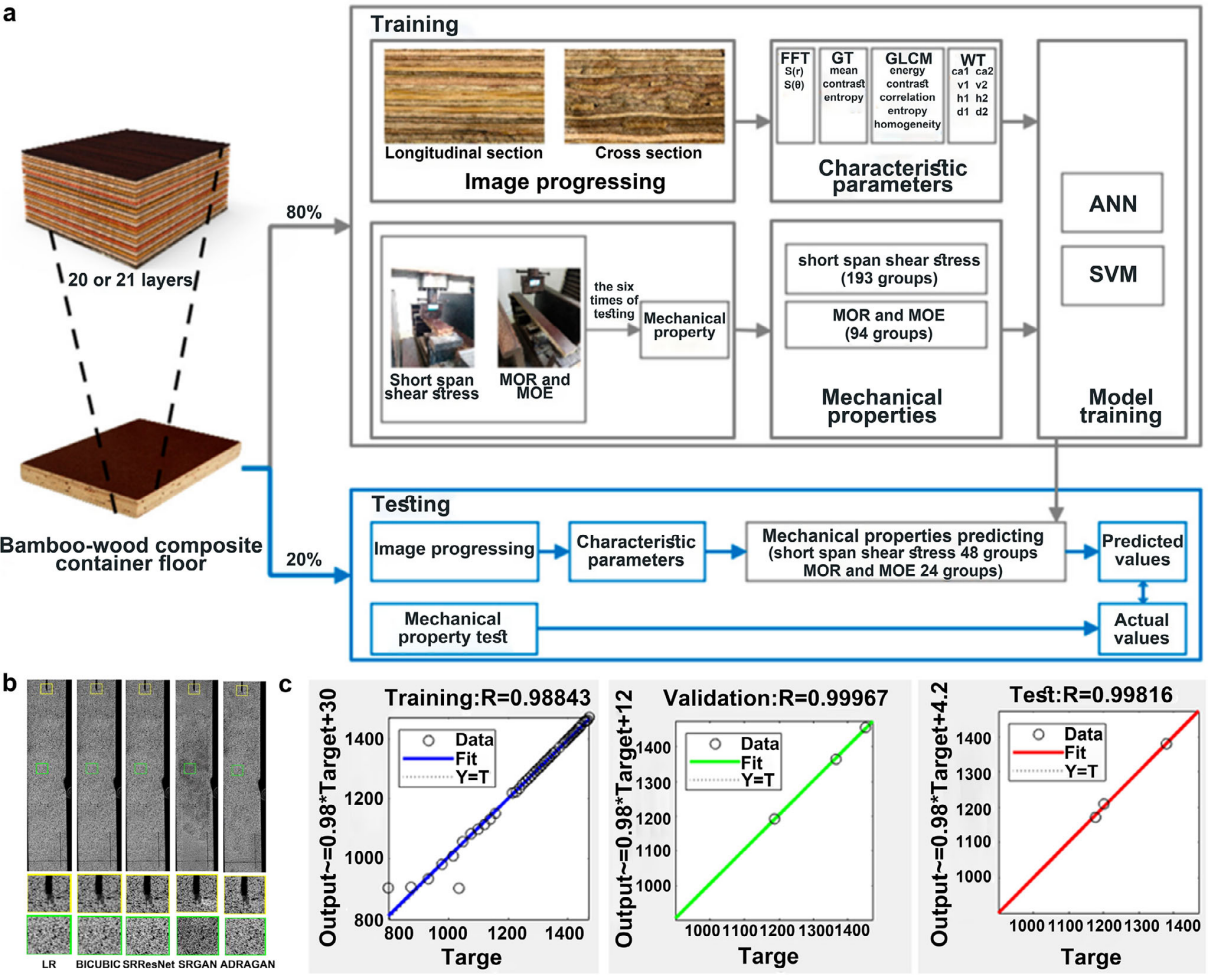
a) Nondestructive testing of mechanical properties of bamboo–wood composite container floor by image processing. Reproduced with permission.^[^ ^201^ ^]^ Copyright 2021, MDPI. b) Comparison of the image reconstruction effects for each algorithm. Reproduced with permission.^[^ ^203^ ^]^ Copyright 2021, MDPI. c) Validation performance of train, validation, and test data. Reproduced with permission.^[^ ^204^ ^]^ Copyright 2024, IAES.

*Predicting mechanical properties of bamboo materials*. Studies have demonstrated the effectiveness of ML models in predicting key properties such as tensile strength, compressive strength, elastic modulus, and stiffness.^[^ ^197^, ^198^ ^]^ The fundamental principle behind these applications is the ability of ML algorithms, such as artificial neural networks (ANNs), to learn complex, non‐linear relationships from data. For instance, an ANN can be trained on a dataset where the inputs are material features (e.g., density, fiber distribution) and the outputs are measured mechanical properties. The model learns a “function” that maps inputs to outputs without being explicitly programmed with physical laws. Algorithms like ANN and extreme learning machines (ELM) have been employed for these purposes, with ELM outperforming other models in predicting tensile and compressive strength based on material features such as density, wall thickness, and fiber distribution. For composite materials like bamboo fiber‐reinforced concrete and laminated bamboo composites, hybrid approaches that combine classical methods like finite element modeling or laminate theory with ML have been effective in capturing complex behaviors, such as ply orientation effects and nonlinear stiffness phases.^[^ ^199^, ^200^ ^]^ Recent efforts have also integrated nondestructive testing methods into ML workflows, enabling efficient and accurate predictions for bamboo‐wood composites^[^ ^201^, ^202^ ^]^ (**Figure** **13**a).

*Process optimization and quality control*. AI applications extend to process monitoring and quality control in bamboo material manufacturing. For optimization, AI can explore a vast parameter space (e.g., pressing temperature, time, resin content) far more efficiently than physical trial‐and‐error, identifying conditions that maximize performance. For quality control, non‐destructive testing methods like image analysis can be used to extract features from a material, which are then fed into a trained ML model to predict its mechanical properties in real‐time. For instance, the application of generative adversarial networks (GANs)—specifically an Attention‐Dense Residual GAN (ADRAGAN)—improved crack detection in engineered bamboo by enhancing image resolution during digital image correlation testing^[^ ^203^ ^]^ (Figure 13b). In composite materials, AI has also been employed to optimize processing conditions (Figure 13c), such as tuning the bamboo–polymer composition to maximize performance metrics like stiffness and modulus. For example, experiments have shown that integrating AI with experimental methods can improve the elastic modulus of bamboo‐reinforced polypropylene composites to 550.3 MPa, optimizing both performance and efficiency.^[^ ^204^ ^]^

Despite these advancements, challenges such as small dataset sizes (e.g., 30–600 samples), limited model generalizability, and underutilization of advanced techniques like transfer learning remain. Furthermore, bamboo's anatomical and mechanical properties vary significantly across species, growth conditions, and processing methods, requiring advanced algorithms to address this variability. Future applications of ML/AI in bamboo‐based materials should include expanding to automated process optimization, functional material design (e.g., predicting antimicrobial or UV‐resistant coatings), and multi‐objective optimization frameworks to simultaneously improve ecological and mechanical performance.

## 5 The Exploration of Circular Economy of Bamboo‐Based Materials

**Figure 14:**
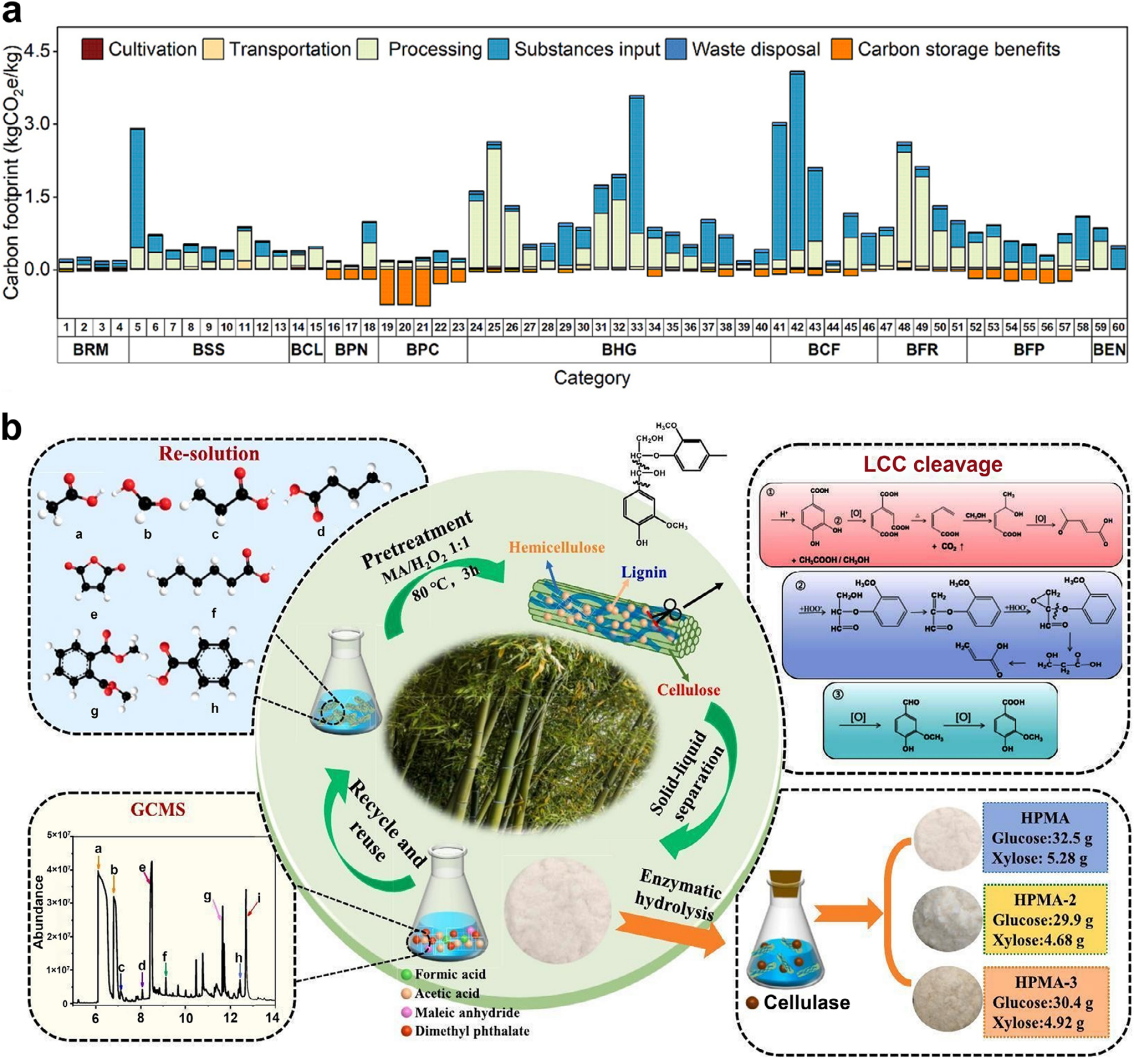
a) Cradle‐to‐grave life cycle carbon footprint of 60 bamboo products. BRM represents bamboo raw materials; BSS represents bamboo shoots; BCL represents bamboo charcoal; BPN represents bamboo‐based panels; BPC represents bamboo panels for construction; BHG represents bamboo house goods; BCF represents bamboo crafts; BFP represents bamboo furniture; BEN represents bamboo extraction, and BFR represents bamboo fiber products. Reproduced with permission.^[^ ^205^ ^]^ Copyright 2025, Elsevier B.V. b) Separation of bamboo cell wall components via H_2_O_2_‐malic acid pretreatment for a waste‐free, integrated biomass biorefinery process. Reproduced with permission.^[^ ^209^ ^]^ Copyright 2025, Elsevier B.V.

The exploration of a circular economy for bamboo‐based materials focuses on creating closed‐loop systems that maximize resource efficiency, minimize waste, and extend product lifecycles. Bamboo, as a renewable, fast‐growing, and carbon‐sequestering resource, offers considerable potential in aligning with sustainability objectives. Studies have emphasized bamboo's lifecycle advantages,^[^ ^205^ ^]^ with life cycle assessment highlighting bamboo's potential for reducing overall carbon footprints.^[^ ^206^, ^207^ ^]^ The carbon footprint of bamboo products ranges from −0.55 to 4.02 kgCO_2_e kg^−1^, with an average value of 0.86 kgCO_2_e kg^−1^. The average carbon storage benefit of bamboo products is −0.10 kgCO_2_e kg^−1^, which offsets 11.78% of carbon emissions^[^ ^208^ ^]^ (**Figure** **14**a). Modular and disassemble bamboo designs in construction (e.g., laminated bamboo panels, cross‐laminated bamboo) facilitate reusability and recyclability in secondary applications. Such designs reduce waste in construction cycles and optimize material recovery at the end of life.

Additionally, bamboo byproducts such as sawdust, lignin, and cellulose can be valorized into high‐value products like biochar, biopolymers, and biofuels through methods such as pyrolysis and enzymatic hydrolysis.^[^ ^209^, ^210^, ^211^ ^]^ For instance, hydrogen peroxide‐malic acid pretreatment enables holistic fractionation of bamboo waste within a zero‐waste closed‐loop biorefinery system, significantly advancing the sustainable utilization of bamboo resources^[^ ^209^ ^]^ (Figure 14b). Biochar derived from bamboo has shown potential as a carbon‐sequestering agent and as a green adsorbent for water and air purification.^[^ ^211^ ^]^ When compared to biochar from other precursors, bamboo‐derived variants often exhibit competitive or superior properties. For carbon sequestration, bamboo's rapid growth cycle and high biomass yield mean that converting it into stable biochar offers a highly efficient pathway for long‐term carbon fixation, potentially surpassing slower‐growing wood species in terms of carbon sequestered per unit time. For adsorption applications, its advantages are particularly evident. For instance, activated carbon derived from bamboo can achieve an ultrahigh specific surface area (SSA) exceeding 2500 m^2^ g^−1^. This is significantly higher than that of many conventional wood‐based biochars (typically 500–1500 m^2^ g^−1^) and is comparable to some high‐performance petrochemical‐derived activated carbons. This superior SSA endows bamboo‐based adsorbents with a distinct advantage in capturing pollutants and for other high‐surface‐area applications. However, challenges persist in fully realizing circularity, as many bamboo products rely on synthetic adhesives, limiting recyclability, though bio‐based adhesives and modular design approaches are emerging as potential solutions.^[^ ^212^ ^]^ Innovations such as binder‐free composites and bamboo fiber‐reinforced materials also contribute to enhanced durability and waste reduction. Despite these advancements, most existing research addresses only isolated aspects of circularity, with limited system‐level integration or real‐world demonstrations of closed‐loop systems. Future efforts must focus on scaling technologies, improving end‐of‐life strategies, and integrating industrial symbiosis models to fully realize the circular economy potential of bamboo.

## 6 Conclusion

This review has provided a focused and critical analysis of recent progress in the development of functional materials and devices derived from bamboo, moving beyond a generalized literature review to explore the diverse potential of specific bamboo components. We emphasized a structure‐property‐function perspective, demonstrating how the unique bamboo hierarchical architecture, cell wall chemistry, and bioactive compounds to their diverse applications. Unlike many previous reviews, this study highlighted species‐specific variations, demonstrating their impact on material performance and emphasizing the necessity of considering this when deriving bamboo‐based materials and devices.

However, realizing the full potential of bamboo for advanced materials applications requires addressing several key challenges. First and foremost, a more fundamental understanding of the relationships between hierarchical structure and performance is still needed, moving beyond bulk characterization and focusing on a multiscale approach that considers the natural variations and anisotropies of bamboo, as well as their species‐specific traits. This includes developing more accurate methods for characterizing and quantifying structural features at different scales and understanding how they contribute to material performance. Second, processing of bamboo materials must become more sustainable, requiring novel approaches and a critical assessment of current methods. Specifically, replacing toxic chemicals with green alternatives, minimizing the consumption of energy, and reducing water usage during processing, are critical for achieving sustainability. Third, the issues related to scalability, long‐term stability, and performance variability of bamboo‐based materials, must be tackled through improved manufacturing processes and specific materials design approaches that enhance component interconnectivity and long‐term structural integrity. For example, developing methods for creating uniform pore structures in bamboo‐based materials is important for controlling their properties for various applications. Finally, computational modeling, while a valuable tool, requires further development to become a more accurate representation of bamboo's complexity. This includes focusing on predicting key properties such as water transport and optical response, as well as focusing on standardized methods that can easily be used across the board. Furthermore, establishing robust parameter sets for different bamboo species will be essential for reliable and predictive modeling.

The future of bamboo material research will depend on targeted studies designed to address these challenges. This requires a synergistic approach, integrating advanced characterization, process optimization, novel computational design, and a greater focus on long‐term material performance. The emphasis must shift from simply utilizing bamboo, to understanding its unique traits and adapting them to specific needs. A focus on exploiting the inherent strengths of each specific bamboo component through innovative processing and design, with an increasing understanding of species‐specific properties, will lead to new opportunities in diverse fields. Ultimately, this review emphasizes the necessity to shift to a more bio‐based economy that prioritizes sustainable practices. The strategic and holistic utilization of bamboo, a renewable and versatile bioresource, can be a driving force for technological advancements, contributing to environmental sustainability and economic growth. The development and widespread adoption of bamboo‐based functional materials have the potential to create new industries, generate employment, and help achieve a more sustainable and equitable world.

## Conflict of Interest

The authors declare no conflict of interest.

## Author Contributions

Y.X.H., W.J.Y. developed the concept. All authors participated in the literature analysis and the processes of figures and visualizations. The work was supervised by D.H.Z., B.B.X., and W.J.Y. The manuscript was written by Y.X.H., D.H.Z., S.B.G., X.M.H., Q.M.J., M.W.S., S.D., B.B.X and W.J.Y., with contributions from all authors.
